# Antitumor activity of the protein kinase inhibitor 1-(β-D-2′-deoxyribofuranosyl)-4,5,6,7-tetrabromo- 1*H-*benzimidazole in breast cancer cell lines

**DOI:** 10.1186/s12885-022-10156-8

**Published:** 2022-10-15

**Authors:** Mirosława Koronkiewicz, Zygmunt Kazimierczuk, Andrzej Orzeszko

**Affiliations:** 1 Department of Biomedical Research, National Medicines Institute, Chełmska St. 30/34, 00-725 Warsaw, Poland; 2 Institute of Chemistry, Warsaw University of Life Sciences, Nowoursynowska St. 159C, 02-787 Warsaw, Poland

**Keywords:** 1-(β-D-2′-deoxyribofuranosyl)-4,5,6,7-tetrabromo-1*H-*benzimidazole, Breast cancer cell lines, Protein kinase inhibitor, Apoptosis, Flow cytometry

## Abstract

**Background:**

The protein kinases CK2 and PIM-1 are involved in cell proliferation and survival, the cell cycle, and drug resistance, and they are found overexpressed in virtually all types of human cancer, including breast cancer. In this study, we investigated the antitumor activity of a deoxynucleoside derivative, the protein kinase inhibitor compound 1-(β-D-2′-deoxyribofuranosyl)-4,5,6,7-tetrabromo-1*H-*benzimidazole (K164, also termed TDB), inter alia CK2 and PIM-1, on breast cancer cell lines (MDA-MB-231, MCF-7, and SK-BR-3).

**Methods:**

An evaluation of the cytotoxic and proapoptotic effects, mitochondrial membrane potential (ΔΨm), and cell cycle progression was performed using an MTT assay, flow cytometry, and microscopic analysis. The Western blotting method was used to analyze the level of proteins important for the survival of breast cancer cells and proteins phosphorylated by the CK2 and PIM-1 kinases.

**Results:**

The examined compound demonstrated the inhibition of cell viability in all the tested cell lines and apoptotic activity, especially in the MCF-7 and SK-BR-3 cells. Changes in the mitochondrial membrane potential (ΔΨm), cell cycle progression, and the level of the proteins studied were also observed.

**Conclusions:**

The investigated CK2 and PIM-1 kinase inhibitor K164 is a promising compound that can be considered a potential agent in targeted therapy in selected types of breast cancer; therefore, further research is necessary.

**Supplementary Information:**

The online version contains supplementary material available at 10.1186/s12885-022-10156-8.

## Background

Worldwide, breast cancer (BC) is the most common cancer in women. BC is routinely classified by stage; pathology; grade; and the expression of estrogen receptor (ER), progesterone receptor (PR), or human epidermal growth factor receptor 2 (HER2). The main treatments for BC are surgery, radiotherapy, chemotherapy, hormone therapy, and targeted therapy, as well as combination of these methods. A potential therapeutic target for many cancers, including breast cancer, is constitutively active serine/threonine kinases: casein kinase 2 (CK2) and the proviral integration site for Moloney murine leukemia virus-1 (PIM-1) [1–6]. Protein kinase CK2 has been implicated in cell growth, proliferation, death, and survival [7, 8]. PIM-1 kinase regulates multiple cellular functions such as the cell cycle, cell survival, and drug resistance [9–14]. Kinases CK2 and PIM-1 have been reported to be overexpressed in solid tumors and hematologic malignancies. The elevation of CK2 and PIM-1 in cancer cells was shown to involve the suppression of apoptosis, suggesting a protective role for these kinases against cell death [1–6, 15–18].

In prostate and lung malignancies, acute myeloid leukemia, pancreatic ductal adenocarcinoma, and breast cancer, overexpression of the kinases CK2 and PIM-1 in patient tumors correlates with poor prognosis and is regarded an unfavorable prognostic marker [19–22]. Elevated PIM-1 expression in invasive breast cancers and benign breast tissue samples was found to be associated with malignancy and a higher tumor grade [23]. In HER2- and hormone-negative cancers, overexpression of PIM-1 is related to a poor prognosis. PIM-1 prevents mitochondrial-mediated apoptosis in triple-negative breast cancer (TNBC) cell lines [24]. It was also demonstrated that kinase PIM-1 may be a potential biomarker for the accurate diagnosis and targeted therapy of TNBC, which is negative for the expression of ER, PR, and HER2, and is associated with a poorer prognosis among all types of BC [25]. CK2 is highly expressed in human breast tumor specimens and in carcinogen-induced rat mammary tumors [26]. The extensive involvement of kinase CK2 in cancer derives from its impact on diverse molecular pathways controlling cell proliferation and cell death [27]. In BC, numerous cell signaling pathways are aberrantly activated to produce the myriad phenotypes associated with malignancy; such pathways include the PI3K/Akt/mTOR, NF-κB, and JAK/STAT cascades. These pathways are highly interconnected, but the kinase CK2 is a prominent lateral enhancer of each [28]. As a result, CK2 has emerged as a viable oncology target having been experimentally validated with different kinase inhibitors, including small-molecule ATP competitors, synthetic peptides, and antisense oligonucleotides [29].

Several classes of inhibitors have been designed to target CK2 with efficacy in low micromolar ranges. These include derivatives of benzotriazole and benzimidazole, e.g., 4,5,6,7-tetrabromo-1*H*-1,2,3-benzotriazole (TBBt) [30], 4,5,6,7-tetrabromo-1*H*-1,2,3-benzimidazole (TBBi), 4,5,6,7-tetrabromo-1*H*-benzimidazole-2-N,N-dimethylamine (DMAT) [31], and one of the most efficient CK2 inhibitors: 4,5,6,7-tetraiodo-1*H-*benzimidazole (TIBI), (Ki = 23 nM) [32]. TBBt, TBBi, DMAT, and TIBI induced apoptosis and cytotoxic effects in leukemia and cancer cell lines [33–37]. DMAT induced cell death in antiestrogen-resistant human breast cancer MCF-7 sublines [38]. A series of polybrominated benzimiadazole derivatives substituted by various cyanoalkyl groups have been synthesized as potential CK2 inhibitors with anticancer and proapoptotic activity [39]. The first orally available small-molecule inhibitor of the CK2 protein in clinical trials for many cancer types was CX-4945 (known as Silmitasertib), (Cylene Pharmaceuticals, San Diego, CA, USA). CX-4945 has shown antitumor activity against other human solid cancer cells such as breast cancer, pancreatic adenocarcinoma, and cholangiocarcinoma. In preclinical studies, antitumor efficacy was investigated in a xenograft model of inter alia breast carcinoma in which CX-4945 showed dose-dependent antitumor activity and reduced the growth of tumors; it was well tolerated at all the doses tested as indicated by the minimal body weight loss and no overt toxicity [40].

Our studies indicate the ability of CK2 inhibitors to enhance the efficacy of 5-fluorouracil (5-FU) in anticancer treatment. We demonstrated that combining CX-4945, 14B, or other inhibitors with 5-FU increased the therapeutic response of the tested breast cancer cell lines [41, 42]. We also described the synthesis of new N-hydroxypropyl TBBi and 2MeTBBi derivatives and their effect on the viability of MCF-7 and MDA-MB-231 cell lines. Derivatives with the methyl group decreased the viability of both cell lines more efficiently than their non-methylated analogs [43].

Additionally, a large number of small-molecule inhibitors of PIM-1 have been developed. The flavonol quercetagetin was identified as a moderately potent, ATP-competitive and a highly selective cell-permeable inhibitor of the PIM-1 kinase [44]. Quercetagetin was shown to inhibit PIM1 activity in prostate cancer cells [44] and to have antiproliferative activity in cervical, breast, and lung cancer cell lines in a dose-dependent manner [45]. SGI-1776, the first PIM-1 inhibitor that targets all three PIM kinases, has been tested in clinical trials in non-Hodgkin lymphoma and prostate cancer patients [46].

**Fig. 1:**
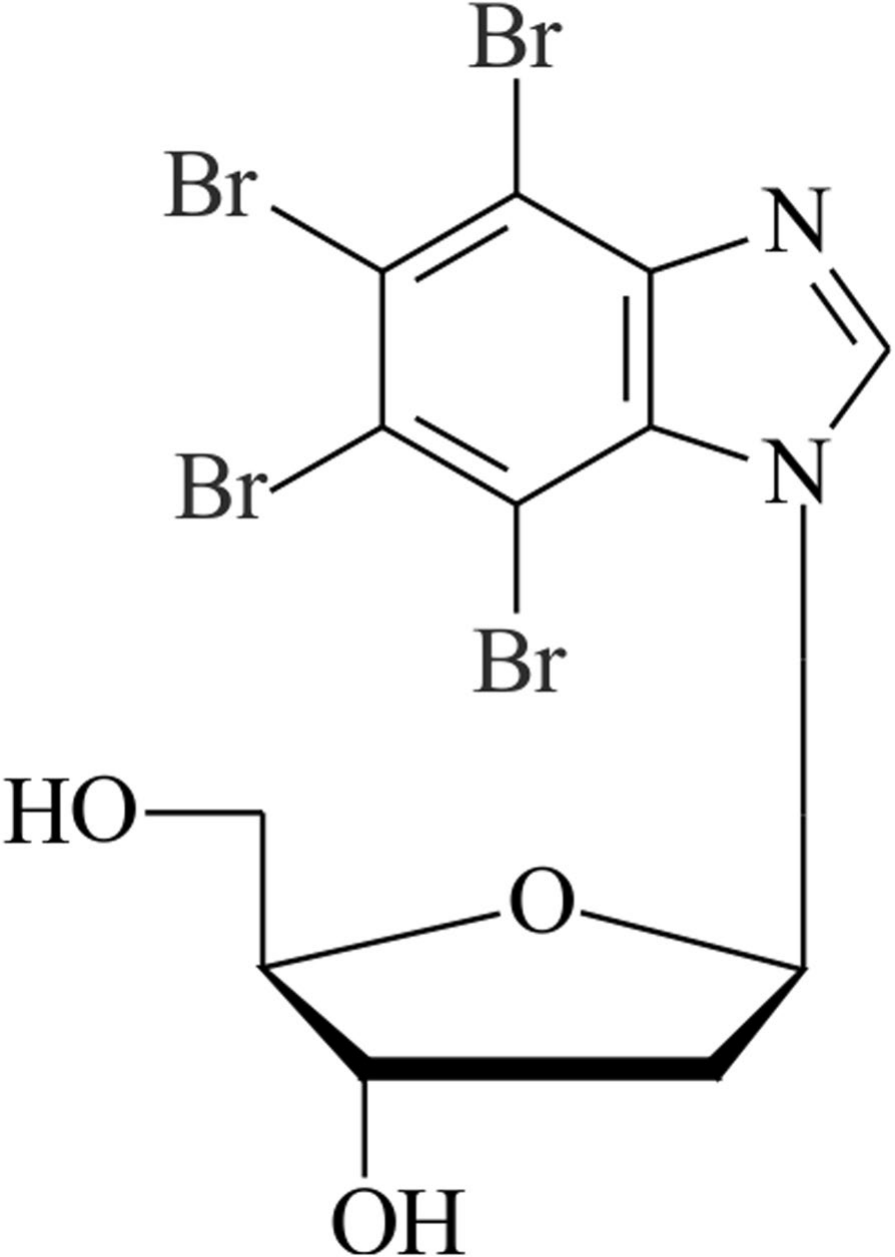
The formula of the studied inhibitor 1-(β-D-2′-deoxyribofuranosyl)-4,5,6,7-tetrabromo-1*H-*benzimidazole (K164, also termed TDB)

In our previous publication, we presented the anticancer activity of deoxynucleosides with various tetrahalobenzimidazoles as an aglycone moiety against neoplastic cell lines in vitro [47]. Our results showed that the tested compounds are potential anticancer agents for targeted therapy, particularly in the treatment of myeloid leukemia and androgen-responsive prostate cancer. Among the compounds studied, dual inhibitors of protein kinases CK2 and PIM-1, compound 1-(β-D-2′-deoxyribofuranosyl)-4,5,6,7-tetrabromo-1*H-*benzimidazole (K164, also termed TDB; Fig. 1), proved to be the most promising [47].

The selectivity and cytotoxic efficacy of the cell-permeable inhibitor K164 was also previously examined by Cozza G. and co-authors [48]. K164, at a 1 μM concentration, was evaluated on a panel of 124 kinases to determine its selectivity. Only CLK2 and DYRK1A were inhibited by K164 as severely as CK2 and PIM-1, as demonstrated. Research has shown that the cell viability was significantly reduced in all of the examined cell lines after 24 hours of treatment with K164, with cancer cells being damaged more drastically than non-tumor cell lines [48].

The above-mentioned results encouraged us to undertake and extend further studies in vitro with the use of the inhibitor K164. In this investigation, we evaluated the anticancer potential of K164 against three BC cell lines with different receptor expressions: ER, PR, and HER2, as well as the status of the p53 protein.

## Methods

### Chemistry

The compound deoxynucleoside derivative protein kinase inhibitor 1-(β-D-2′-deoxyribofuranosyl)-4,5,6,7-tetrabromo-1*H-*benzimidazole (K164) was synthesized using the same procedure as described previously [48, 49].

### Cell culture and treatment with inhibitor

Human breast carcinoma cell lines were used in this study, which differ in their presence/absence of receptors: ER, PR, HER2, and the status of protein p53, namely, triple-negative breast cancer (TNBC) MDA-MB-231 (ER−/PR−/HER2−/mutant p53), MCF-7 (ER+/PR+/HER2−/p53-wild type), and SK-BR-3 (ER−/PR−/HER2+/mutant p53). The MDA-MB-231 and MCF-7 cell lines were purchased from the American Type Culture Collection (ATCC, Manassas, VA, USA) and SK-BR-3 from German Collection of Microorganisms and Cell Cultures GmbH (DSMZ, Braunschweig, Germany). The MDA-MB-231 cells were grown in Iscove’s Modified Dulbecco’s medium with stable glutamine (Biowest, Nuaillé, France) supplemented with 10% (v/v) heat-inactivated fetal bovine serum (Biowest), 1% (v/v) MEM non-essential amino acids (Biowest), and 1% (v/v) antibiotic–antimycotic solution (Biowest). The MCF-7 cells were grown in Eagle’s MEM (Minimal Essential Medium) medium with stable glutamine (Biowest), supplemented with 10% (v/v) heat-inactivated fetal bovine serum (Biowest), 1% (v/v) MEM non-essential amino acids (Biowest), 1% (v/v) antibiotic–antimycotic solution (Biowest), and 1 mM sodium pyruvate (Sigma-Aldrich, St. Louis, MO, USA). The SK-BR-3 cells were grown in McCoy’s 5A medium (Biowest) supplemented with 10% (v/v) heat-inactivated fetal bovine serum (Biowest), 1% (v/v) MEM non-essential amino acids (Biowest), 1% (v/v) antibiotic–antimycotic solution (Biowest), and 1% (v/v) stable glutamine (L-alanyl-L-glutamine) solution (Biowest). The cell lines were cultured at 37 °C in a humidified atmosphere of 5% CO_2_ in air. All the experiments were performed in exponentially growing cultures. The compound studied was added to the cultures as solutions in dimethyl sulfoxide (DMSO) (Sigma-Aldrich, St. Louis, MO, USA); control cultures were treated with the same volume of the solvent. The final concentration of DMSO was maintained at ca. 0.1%. The cells were collected and labeled after being incubated (24 or 48 hours) with the examined compound.

### Cell viability (MTT colorimetric assay)

Cell viability was assessed using 3-(4,5-dimethylthiazol-2-yl)-2,5-diphenyltetrazolium bromide (MTT) (Sigma-Aldrich). The cells were cultured in 96-well plates and incubated for 24 h or 48 h with the tested compound. MTT stock solution was added to each well to a final concentration of 0.5 mg/ml and incubated for 4 h at 37 °C, next formazan crystals were dissolved by the addition of SDS-HCl solution (10% SDS in 0.001 M HCl, final concentration). MTT and SDS were added directly to the cell culture. The solubilized formazan product was spectrophotometrically quantified in a Power Wave XS (Bio Tek, Winooski, VT, USA) microplate reader at a wavelength of 570 nm. The IC_50_ values (concentration required to reduce the viability of cells by 50% compared with the control cells) of the compound were calculated from the data obtained with the MTT assay. Regression analysis was performed using the SigmaPlot software (San Jose, CA, USA).

### Morphological evaluation (inverted microscopy)

The cells’ morphology was evaluated using an ITM-2 inverted microscope equipped with a DP10 digital camera (Olympus, Japan).

### Apoptosis assay by Annexin V/propidium iodide (PI) labelling

The FITC Annexin V Apoptosis Detection Kit I (BD Pharmingen) was used to measure apoptosis. The cells were collected by centrifugation after a 24 or 48-hour incubation with the tested agent, washed twice with cold phosphate-buffered saline (PBS), and suspended in binding buffer at 1 × 10^6^ cells/ml. Then, 100 μl aliquots of the cell suspension were labelled according to the kit manufacturer’s instructions. The Annexin V-FITC and PI were added to the cell suspension, and the mixture was vortexed and incubated for 15 min at room temperature in the dark. Next, 400 μl of cold binding buffer was added, and the cells were vortexed again and kept on ice. The samples were analyzed using a flow cytometry within 1 hour of labeling.

### Mitochondrial membrane potential (ΔΨm) assay

Mitochondrial membrane potential was assessed by flow cytometry using JC-1 (5,5′,6,6′-tetrachloro-1,1′,3,3′-tetraethylbenzimidazolocarbocyanine iodide; Sigma). In mitochondria, JC-1 accumulates in a potential-dependent manner. In healthy cells, the dye accumulates in the mitochondria, forming aggregates with red fluorescence (FL-2 channel), whereas, in apoptotic cells, the dye remains in the cytoplasm in a monomeric form and emits green fluorescence (FL-1 channel). After experiment (48 h) the cells were harvested by centrifugation, suspended in 1 ml of complete culture medium at approximately 1 × 10^6^ cells/ml, and incubated with 2.5 μl of JC-1 solution in DMSO (1 mg/ml) for 15 min at 37 °C in the dark. The stained cells were washed with cold PBS, suspended in 400 μl of PBS, and then examined by flow cytometry and analyzed using the FACSDiva (BD Biosciences, San Jose CA, USA) and WinMDI 2.8 (Joseph Trotter) software.

### Western blot analyses

The cells were washed with cold PBS buffer, and then whole-cell extractions were prepared using M-PER reagent (Pierce, Rockford, IL, USA). The protein concentration in the samples was measured using a BCA protein assay kit (Pierce). Equal amounts of proteins were loaded on 8% or 10% SDS-PAGE gels. The proteins were transferred to a nitrocellulose membrane after electrophoresis and probed with primary antihuman antibodies specific for the proteins to: BAD, BCL-2, XIAP, p53 (Apoptosis I Sampler Kit) (BD Biosciences), PARP (113 kDa) (Apoptosis II Sampler Kit) (BD Biosciences), PARP (89 kDa) (BD Biosciences), Akt-1, phospho-Akt1 (Ser129), phospho-Bad (Ser112), phospho-p53 (Ser15) (Cell Signaling Technology, Danvers, MA, USA), and phospho-p53 (Ser392) (Thermo Fisher Scientific); secondary antibodies conjugated with HRP anti-mouse (BD Pharmingen) and HRP anti-rabbit (Cell Signaling Technology) were used. To visualized of protein bands were use the SuperSignal West Pico PLUS Chemiluminescent Substrate (Thermo Fisher Scientific, Rockford, IL, USA). Signals were detected by chemiluminescence detection system MicroChemi (Bio-Imaging Systems).

### Cell cycle analysis

After exposure to K164, the cells were washed with cold PBS and fixed in 70% ethanol for at least 24 hours at − 20 °C. Next, the cells were washed in PBS and stained with 50 μg/ml PI and 100 μg/ml RNase solution in PBST (PBS supplemented with 0.1% v/v Triton X-100) for 30 min of incubation in the dark at room temperature. Flow cytometry employing BD FACSDiva software was used to measure cell DNA content. Distribution of cells in different phases of the cell cycle was analyzed by MacCycle software (Phoenix Flow Systems, San Diego, CA, USA).

### Statistical analysis

The data are presented as the mean values ± SDs (standard deviations). Statistical comparisons among groups were performed using Student’s t-test. Significance was assumed at *p* < 0.05 (marked with asterisks).

## Results

### Cytotoxic activity

In BC cell lines, the cytotoxic activity of the deoxynucleoside derivative protein kinase inhibitor compound K164 (Fig. 1) was determined by assessing the number of viable cells using an MTT assay. The results obtained in the test were analyzed to calculate the IC_50_ values of the compound (Table 1). The compound showed weak cytotoxic activity in the studied cells after the 24 h incubation time, while the 48 h incubation induced a stronger effect. K164 was the most active against the SK-BR-3 cell line, and the IC_50_ value was 8.92 μM at 48 h of incubation time.

**Table 1:** IC_50_ values of compound studied (MTT assay)

	IC_50_ [μM]	
Cell line	24 h	48 h
MDA-MB-231	61.93 ± 7.15	27.00 ± 4.10
MCF-7	56.04 ± 6.88	23.67 ± 5.70
SK-BR-3	59.46 ± 6.54	8.92 ± 2.08

### Changes in cell morphology

**Fig. 2:**
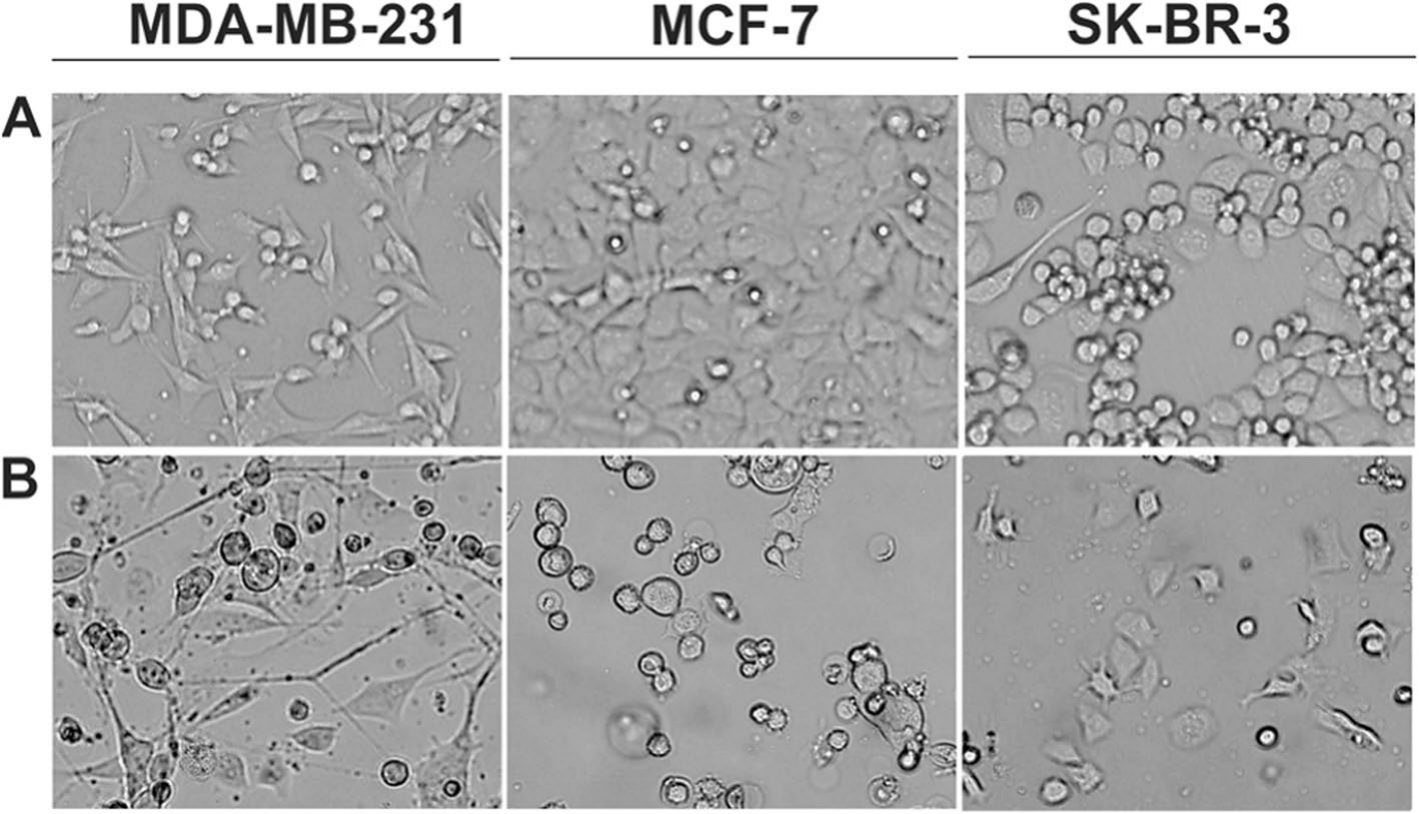
The morphology of breast cancer cells cultured for 48 h, inverted microscopy. Panel **A** Control cells. Panel** B** Cells treated with compound (10 μM)

**Fig. 3:**
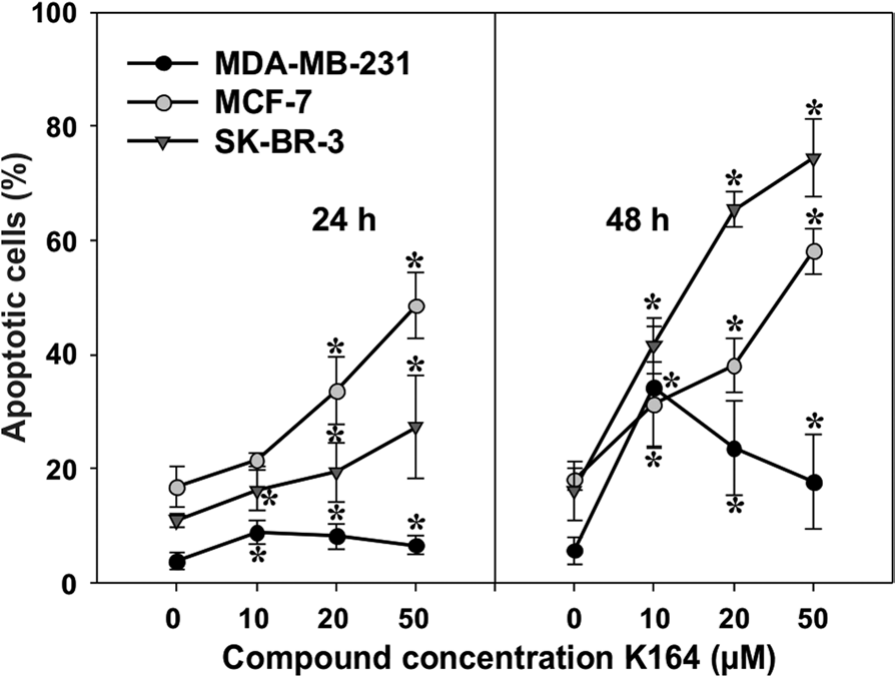
Induction of apoptosis (sum of early and late apoptosis) by the inhibitor in BC cells. The data were determined by a FACS cytometer after 24 and 48 h of treatment. Cells were stained with Annexin V-FITC and PI. Each point represents the mean ± S.D.; asterisks indicate significance at *p* < 0.05 for comparison with the control

The effects of the compound on the morphology of cells were observed by inverted microscopy. In Fig. 3, we present only the chosen representative pictures. No changes were observed for the untreated cells; however, already at the lowest concentration used, i.e., 10 μM, large changes in the cell morphology were observed, demonstrating the inhibition of proliferation of all the tested cell lines (Fig. 2).

### Induction of apoptosis in cell lines

Apoptosis was determined using Annexin V (FITC)/PI labeling of the cells after 24 and 48 h of incubation with the compound. The examined inhibitor induced apoptotic death in cells; among the tested cell lines, the most sensitive were the SK-BR-3 cells. The exemplary concentration of 20 μM K164 and incubation times of 24 h and 48 h evoked 19.42 and 65.38% apoptosis in the SKBr-3 line; in the MCF-7 line, this was 33.65 and 38.06%; and in the MDA-MB-231 line, it was 8.22 and 23.63%, respectively (Fig. 3).

### Changes in mitochondrial membrane potential (ΔΨm)

**Fig. 4:**
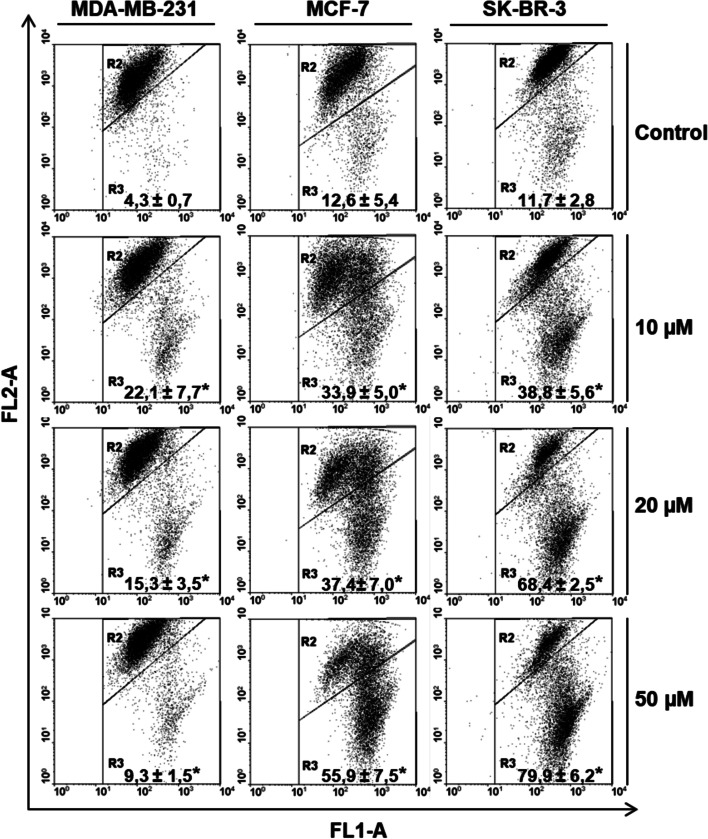
Representative flow cytograms demonstrating changes in mitochondrial membrane potential (ΔΨm) of cells incubated with the K164 for 48 h. The cells were stained with JC-1 dye. The cells in the lower right region (R3) showed increased green fluorescence (apoptotic cells). Asterisks indicate significance at *p* < 0.05 for comparison with the control

Apoptosis was confirmed by assessing the mitochondrial membrane potential. Analysis of the flow cytograms (Fig. 4) showed that the tested inhibitor increased mitochondrial membrane depolarization (as evidenced by the shift in the red-to-green fluorescence ratio) in all the cell lines.

### Effect of the compound on the level of proteins

**Fig. 5:**
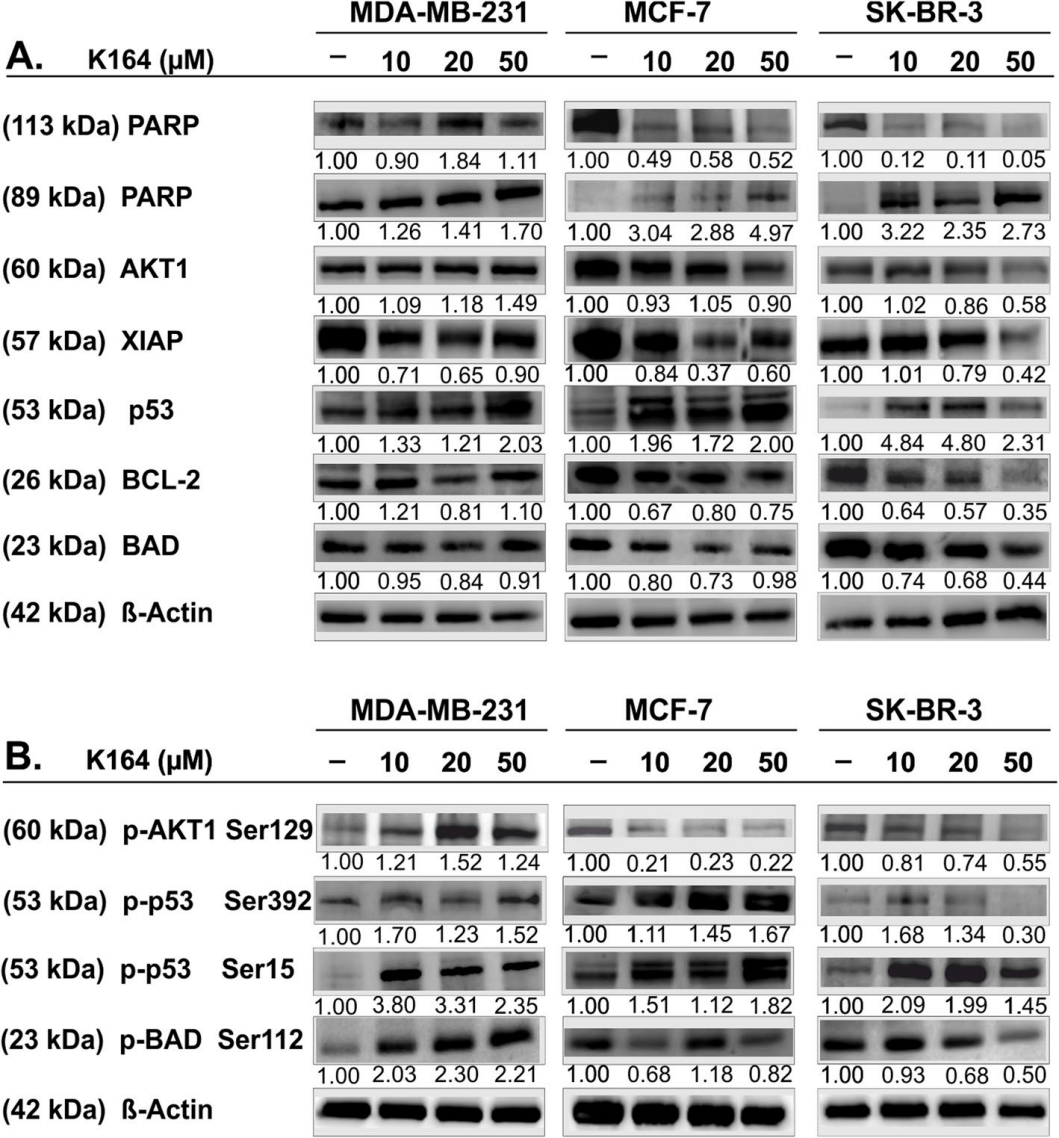
Western blot analysis of proteins in whole cell extracts obtained from cells cultured in the presence of K164 (concentrations of 10 μM, 20 μM, and 50 μM) after 48 h of incubation. Preparation of cell extracts and protein detection are described in Materials and Methods. Densitometry analysis was used to quantify all bands, and their intensity was normalized with respect to β-actin. For cell lines cultured in the absence of K164, the ratio of the examined proteins to β-actin was assumed to be 1. Panel A: PARP, AKT1, p 53, and B-cell lymphoma 2 (BCL-2) family proteins (pro- and anti- apoptotic). Panel B: phosphorylated proteins

We performed a Western blot analysis to determine the level of proteins involved in the process of apoptosis, as well as the proteins that are phosphorylated by kinase CK2 (Akt1, p53) and PIM-1 (Bad). This analysis was conducted for whole-cell extracts obtained from cells cultured in the presence of the inhibitor K164 (concentrations of 10 μM, 20 μM, 50 μM) after 48 h of incubation. We revealed that the levels of proapoptotic (Bad) and anti-apoptotic (BCL-2, XIAP) proteins decreased after treatment with K164 in the MCF7 and SK-BR-3 cell lines (Fig. 5). In the MDA-MB-231cells treated with the inhibitor, the levels of BAD and XIAP were decreased, while the BCl-2 protein level increased slightly. Characteristic changes were noticed in level of PARP proteins (113 kDa and 89 kDa). In cell lines undergoing apoptosis (SK-BR-3 and MCF-7), we observed a decrease in the level of total PARP (113 kDa) and an increase in the level of the cleaved PARP protein (89 kDa). The changes in the level of these proteins in the MDA-MB-231 cells were the smallest. The level of the p53 protein was increased in all the cell lines after treatment with the inhibitor. There was a decrease in the level of the AKT protein in the MCF-7 and SK-BR-3 cells, except the MDA-MB-231 cell line. Additionally, the level of phosphorylated proteins Akt1 (Ser129) and Bad (Ser112) was decreased in the cell lysates obtained after treatment of the MCF-7 and SK-BR-3 cells with an inhibitor, while in the MDA-MB-231 cells, an increased level of these proteins was observed. The levels of phosphorylated proteins p-p53 (Ser15) and p-p53 (Ser392) were increased in cells, but in the SK-BR-3 cell line, the level of p-p53 (Ser392) decreased after incubation at the highest concentration of the inhibitor. Figure 5 shows an exemplary plot of a Western blot analysis.

### The compound’s impact on cell cycle progression

**Fig. 6:**
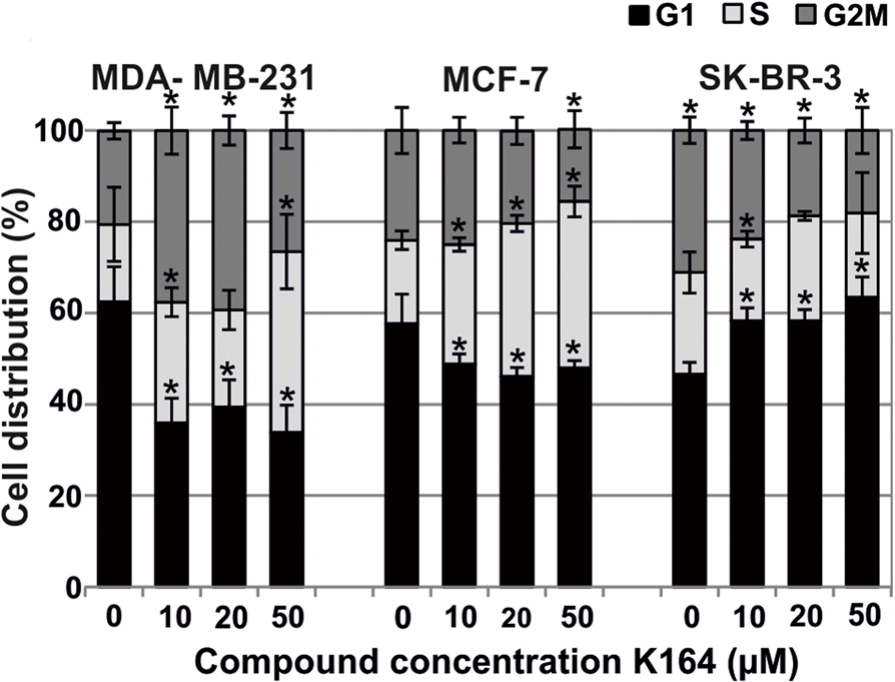
Changes in cell cycle progression in cells after 48 h treatment with K164. Each bar represents the mean ± S.D. (*n* ≥ 5). The data obtained from FACS Canto II flow cytometer (BD Biosciences, San Jose CA, USA) were analyzed using MacCycle software to determine the percentage of cells in each phase of the cell cycle. Each bar represents the mean ± SD; asterisks indicate significance at *p* < 0.05 for comparison with the control

**Fig. 7:**
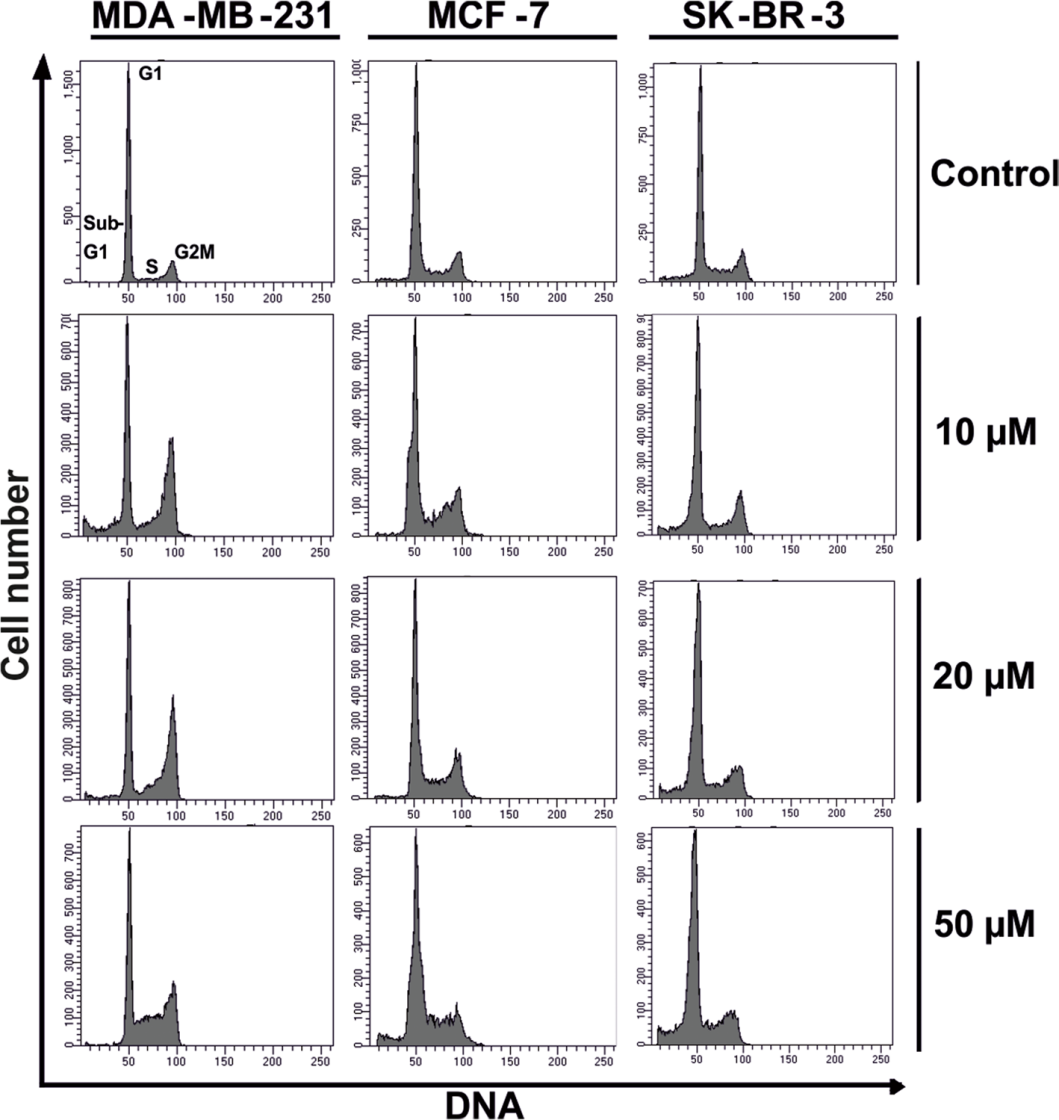
Exemplary DNA histograms of cell lines treated for 48 h with K164 compound (stained with propidium iodide)

Figures 6 and 7 demonstrate changes in the cell cycle progression of cells after 48 h of incubation with the tested compound. The K164 compound exerted a cytostatic effect and caused an accumulation of cells in the G2M and S phases, and, at the border of these phases, a decrease in the number of cells in the G1 phase of the cell cycle in the MDA-MB-231 cells. We observed the accumulation of MCF-7 cells in the S phase and SK-BR-3 cells in the G1 phase.

## Discussion

Protein kinases CK2 and PIM-1 are involved in cell proliferation, survival, the cell cycle, and drug resistance, and they are found overexpressed in virtually all types of human cancer, including BC, hence they are considered a potential target of therapy. In data published previously by us and another group [47–49], it was demonstrated that the inhibitor of protein kinases CK2 and PIM-1 (Fig. 1), 1-(β-D-2′-deoxyribofuranosyl)-4,5,6,7-tetrabromo-1*H-*benzimidazole (K164, also termed TDB), show anticancer activity against leukemia and cancers in vitro.

In this study, we investigated the antitumor activity of inhibitor K164 on BC cells. First, we examined the cytotoxicity of the tested compound on cells by assessing the number of viable cells with an MTT assay (Table 1). K164 demonstrated the inhibition of cell viability, confirmed also by changes in the cell morphology (Fig. 2). The obtained IC_50_ values (Table 1) showed that K164 is the most cytotoxic against breast adenocarcinoma, SK-BR-3 cells. In the next step, we demonstrated apoptotic cell death (Fig. 3), which occurs in a concentration- and time-dependent manner in SK-BR-3 and MCF-7 cells. Parallel changes in the mitochondrial membrane potential were detected (Fig. 4). We observed depolarization of the mitochondrial membrane; this effect was dose-dependent and indicated the intrinsic apoptotic pathway. The MDA-MB-231 cell line showed only slight apoptosis, which was concentration independent but incubation time dependent. In this line, the most effective for the induction of apoptosis was the incubation of cells with 10 μM K164 (Fig. 3). This may be related to the rather high level of the antiapoptotic protein XIAP, especially at a concentration of the compound of 50 μM. Such a phenomenon did not occur in the other two cell lines, where the level of XIAP decreased, similar to the second antiapoptotic protein BCL-2 (Fig. 5). XIAP is a member of the inhibitors of the apoptosis family of proteins (IAPs), which are overexpressed in cancer cells, and it is modulated by CK2 [50]. The expression of BCL-2, an apoptotic cell death suppressor, was studied in 52 invasive breast carcinomas [51]. In this study, related factors such as p53 protein accumulation, hormone receptor status, and apoptotic cell index were also examined. BCL-2 expression correlated significantly with the hormone receptor status, whereas it showed significant inverse correlations with p53 accumulation and the apoptotic index. It was found that estrogen and mutant p53 are linked to BCL-2 expression regulation, and that BC can develop the potential to suppress tumor cell death caused by BCL-2 [51].

Our analysis performed by Western blot of whole-cell extracts confirmed the increase in the p53 level of cells undergoing apoptosis (SK-BR-3 and MCF-7) with decreases in the level of the BCL-2 protein (Fig. 5). We also observed a decreased level of the proapoptotic protein Bad (total) in all three lines and a decreased level of phospho-Bad (Ser112) in the MCF-7 and SK-BR-3 cell lines. The phospho-Bad (Ser112) level in the MDA-MB-231 cells increased significantly after treatment with the inhibitor, which supports cell survival and prevents the occurrence of apoptosis (Fig. 5). The PIM-1 kinase promotes Bad protein inactivation by phosphorylating it on the Ser112 gatekeeper site, as previously documented, and this is one of multiple mechanisms by which PIM-1 kinase can improve BCL-2 activity and support cell survival [52]. The overexpression of PIM-1 in TNBC cell lines prevents mitochondrial-mediated apoptosis [24]. We have shown in our results an increased level of PARP protein cleavage (89 kDa), considered to be a marker of apoptosis, and a decreased level of total PARP (113 kDa) in cells cultured with K164. Akt (also known as PKB) is an anti-apoptotic and pro-survival protein kinase, whose activity is frequently abnormally high in tumors. Three isoforms of Akt exist, and among them, Akt1 and Akt2 are the most widely and highly expressed [53]. Kinase CK2 phosphorylated Akt1 at Ser129 and can also enhance cancer cell survival [54]. We showed that the inhibitor K164 decreased the level of AKT1 only at 50 μM K164 in the MCF-7 and SK-BR-3 cell lines and decreased the level of phospho-AKT1 (Ser129) at all the used concentrations of K164, where the induction of apoptosis was significant, while in the MDA-MB-231 cells, we noted an increase in the level of these proteins, which led to the survival of cells and inhibited apoptosis (Fig. 5). The response to K164 by the tested cells may be related to the expression of the receptors and the p53 protein status. It was founded that, in ER-positive breast tumors, ER represses the p53-mediated apoptotic response induced by DNA damage. The authors of the publication in [55] reported that, in response to doxorubicin-based chemotherapy in BC ER-positive and *TP53* wild-type (WT), the suppression of the p53 apoptotic response by the ER would lead to tumor cell senescence and resistance to treatment. The accumulation of genetic abnormalities in ER-negative *TP53* mutated BC, on the other hand, would lead to a mitotic catastrophe and a better response. Additionally, our results revealed a better response by the SK-BR-3 (ER−/mutant p53) cells to the applied inhibitor in comparison to that of the MCF-7 (ER+/ p53-wild type) cells (Table 1, Figs. 3 and 4). However, the expression of the HER2 receptor is not without significance because HER2-positive breast cancer accounts for 20–25% of all breast cancers [56]. They are aggressive and associated with poor prognosis [57, 58]. HER2-positive cancers also frequently harbor mutations in the *TP53* tumor suppressor gene, which worsens the unfavorable prognosis [59]. HER2 overexpression is induced by p53 mutants through *HER2* transcriptional activation [60]. HER2 is a receptor tyrosine kinase that regulates cell growth, survival, differentiation, and migration. It belongs to the ERBB family of receptor tyrosine kinases. Several HER2-targeted treatments, including tyrosine kinase inhibitors such as lapatinib, neratinib, tucatinib, and pyrotinib have been developed in recent years.

Treatment options involving biologic agents with various mechanisms of action are still being developed. These compounds target a number of intracellular processes involved in the spread of HER2-positive BC [61, 62]. Tumor suppressor p53 plays an important role in cancer prevention. Under normal conditions, p53 is maintained at a low level. Whereas, in response to various cellular stresses, p53 is stabilized and activated, which, in turn, initiates DNA repair, cell cycle arrest, senescence, and apoptosis. Phosphorylation of p53 plays an important role in modulating its activation to induce apoptosis in cancer cells. Several serine/threonine kinases regulate p53 phosphorylation and downstream gene expression [63]. Two different, independent groups were the first to show that the protein kinase CK2 phosphorylates p53 [64, 65]. The data indicate that p53 is a physiological substrate of CK2, which is stimulated in response to mitogens, phosphorylates nuclear oncoproteins, and may play a role in the transduction of extracellular signals to the nucleus. Ser392 is a target of several protein kinases in vitro including CK2 [64, 65]. The penultimate residue in the p53 protein, Ser392 in human p53 (Ser389 in murine p53), was identified as a phospho-acceptor residue, and the association of p53 with CK2 in immunoprecipitation experiments was also demonstrated [66]. Phosphorylation of serine392 in p53 was reported to occur preferentially in response to UV radiation [67] as well as in response to other diverse stimuli [68, 69]. Serine 15 is the primary target of the DNA damage response on the p53 protein and is phosphorylated by both the ATM and ATR protein kinases [70, 71]. In our study, the levels of p53 and the phosphorylated proteins p-p53 (Ser15) and p-p53 (Ser392) in cells generally increased after exposure to the compound, which initiated DNA repair and cell cycle arrest, especially in MDA-MB-231 (Figs. 6 and 7), and induced apoptosis in the MCF-7 and SK-BR-3 cells (Fig. 3). The obtained results encourage us to undertaken to study on K164 in an in vivo model of breast cancer. Research using animal model systems will enable the delivery of improved therapies for breast cancer using a protein kinases inhibitor.

## Conclusions

The investigated CK2 and PIM-1 kinase inhibitor K164 demonstrated different cytotoxic and proapoptotic effects in the studied breast cancer cell lines. Depolarization of the mitochondrial membrane potential was detected in cells after treatment with the compound; this effect indicates the intrinsic apoptotic pathway. Our study revealed the influence of the compound on the cell cycle progression as well the level of proteins involved in the process of apoptosis, as well as proteins that are phosphorylated by kinase CK2 and PIM-1.

To summarize, the researched inhibitor is a promising compound that can be considered a potential agent in targeted therapy in selected types of breast cancer.

## Supplementary Information

**Additional file 1.**

## Data Availability

The datasets used and/o analyses during the current study are available from the corresponding author on reasonable request.
