# Supplementary material for: Antitumor activity of the protein kinase inhibitor 1-(β-D-2′-deoxyribofuranosyl)-4,5,6,7-tetrabromo- 1H-benzimidazole in breast cancer cell lines
Source: BMC Cancer. 2022 Oct 15;22:1069. doi: 10.1186/s12885-022-10156-8 (PMC9571492; doi:10.1186/s12885-022-10156-8)

MDA-MB-231

Western blots shown in Fig. 5

MDA-MB-231_B-Actin

70 kDa
55 kDa
40 kDa

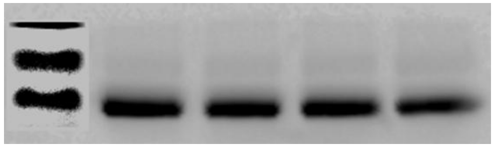

Marker Control 10 20 50 [µM]; 48h

<u>The same order in all blots</u>

PageRuler Prestained Protein Ladder (Marker)

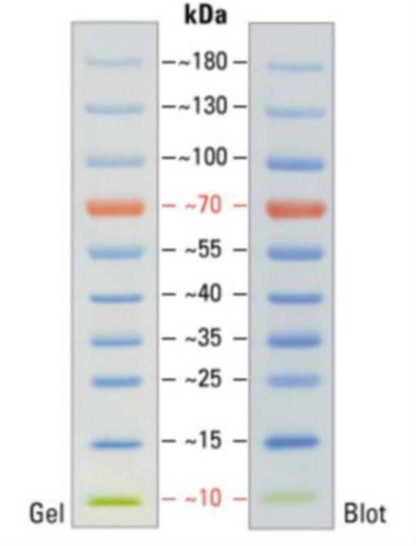

MDA-MB-231_48h_AKT

70 kDa
55 kDa
40 kDa
35 kDa

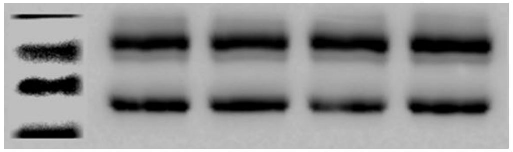

MDA-MB-231_PARP 89

180 kDa
130 kDa
100 kDa
70 kDa

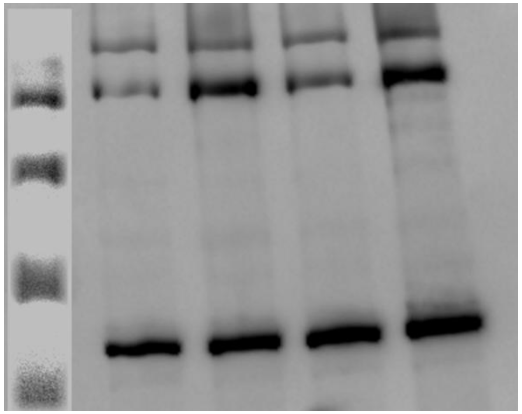

MDA-MB-231_PARP 113

180 kDa
130 kDa
100 kDa
70 kDa

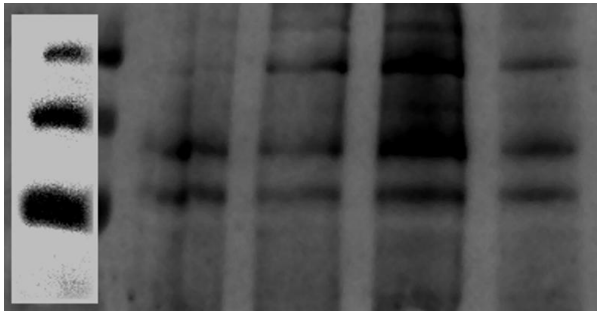

Western blots shown in Fig. 5

MDA-MB-231_BCL-2

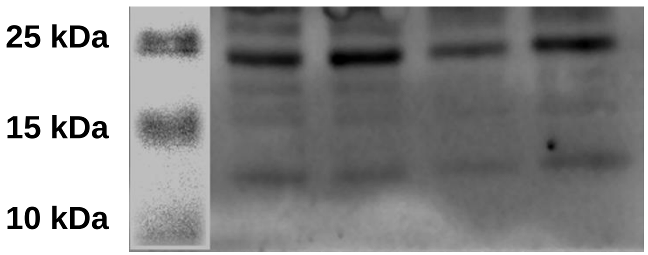

MDA_MB-231_XIAP

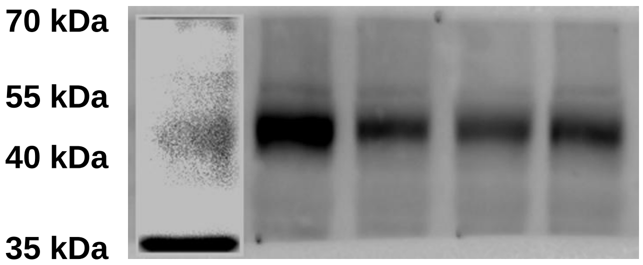

MDA MB-231_BAD

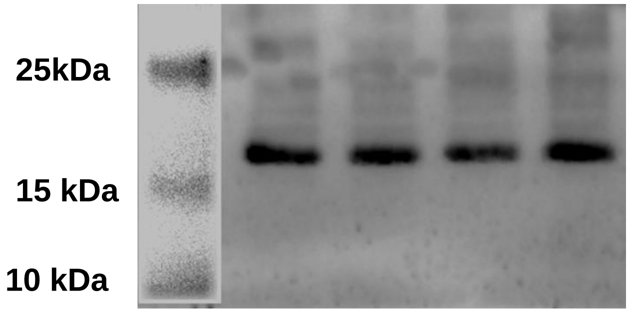

MDA-MB-231_p53

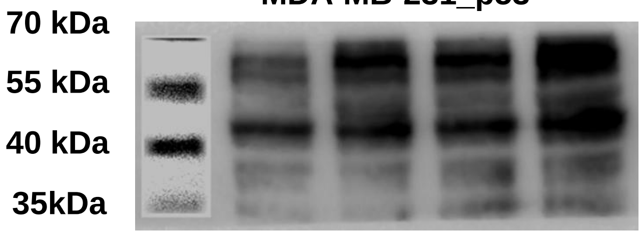

MDA MB-231_24 h_48 h_p-BAD_Ser112

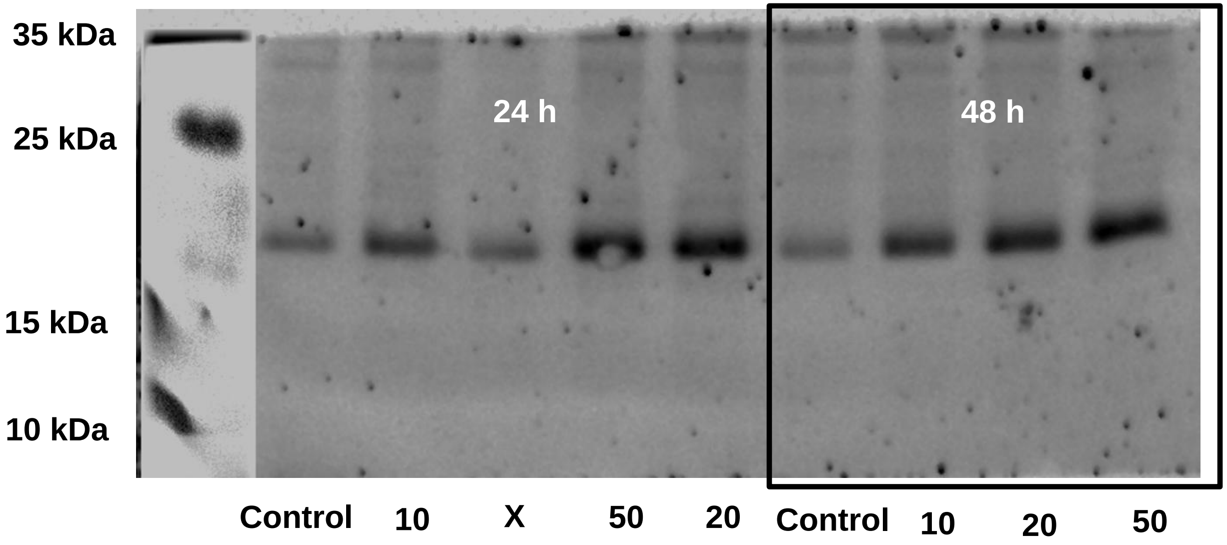

Western blots shown in Fig. 5

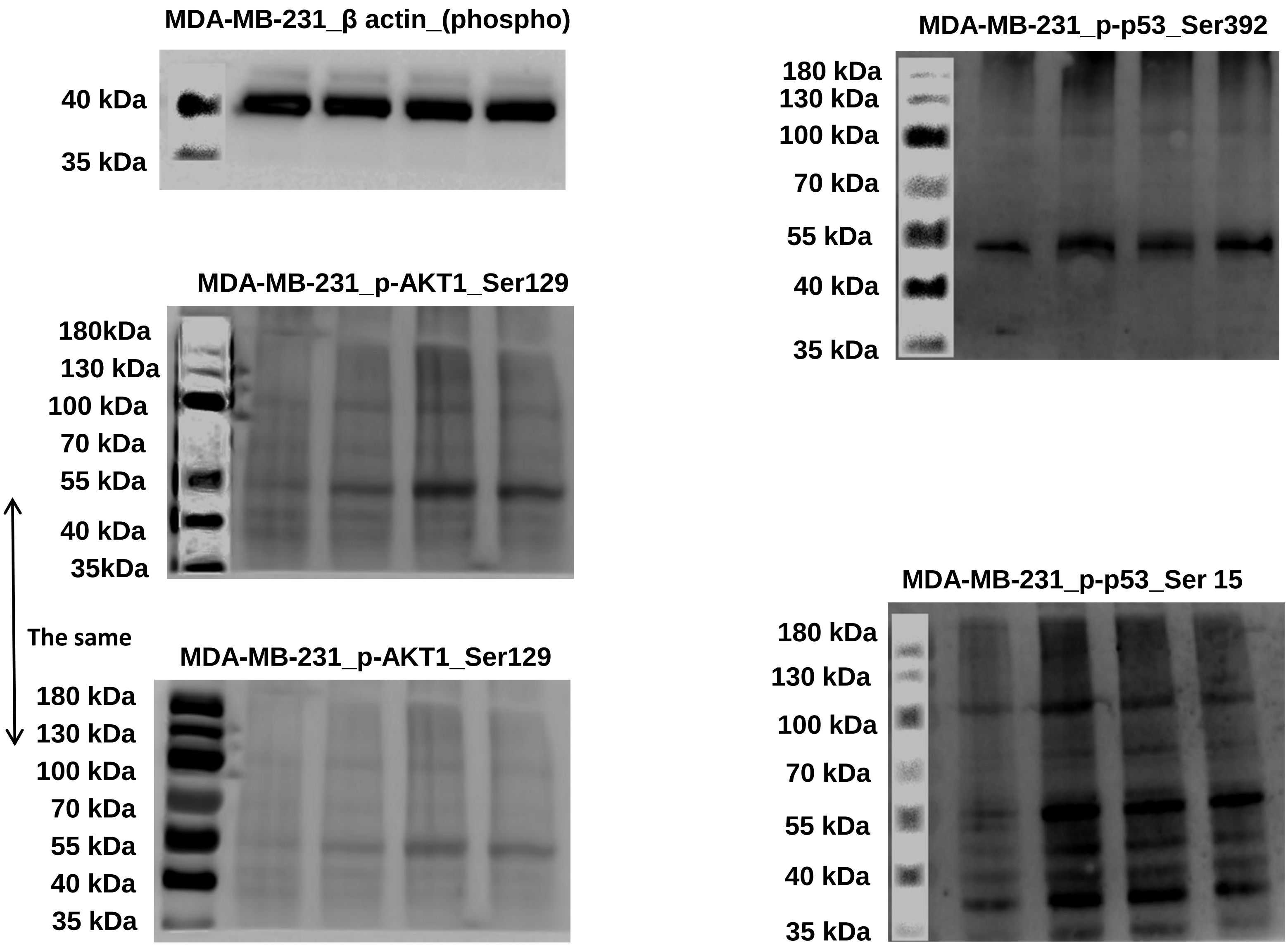

Western blots not shown in Fig. 5 (additional repetitions)

MDA-MB-231_BAD

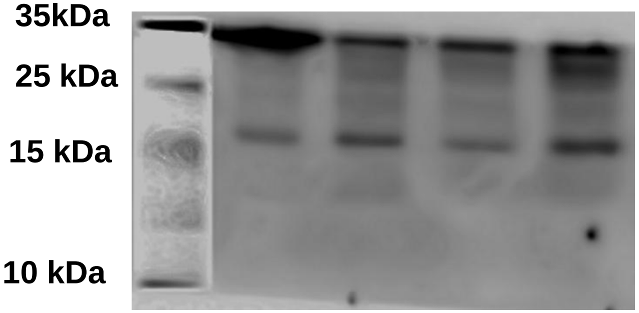

MDA-MB-231_BAD

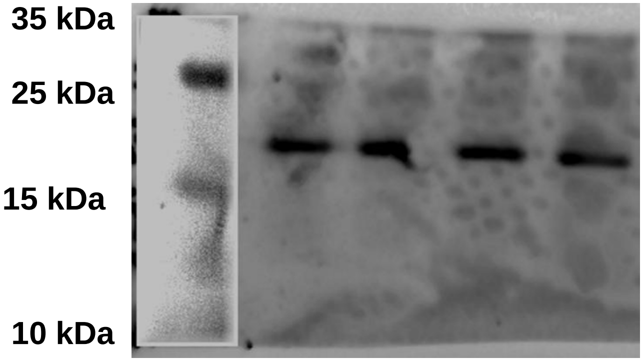

MDA-MB-231_48h_BCL-2

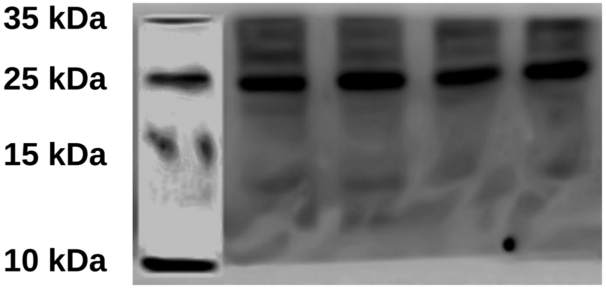

MDA-MB-231_24, 48h_BCL-2

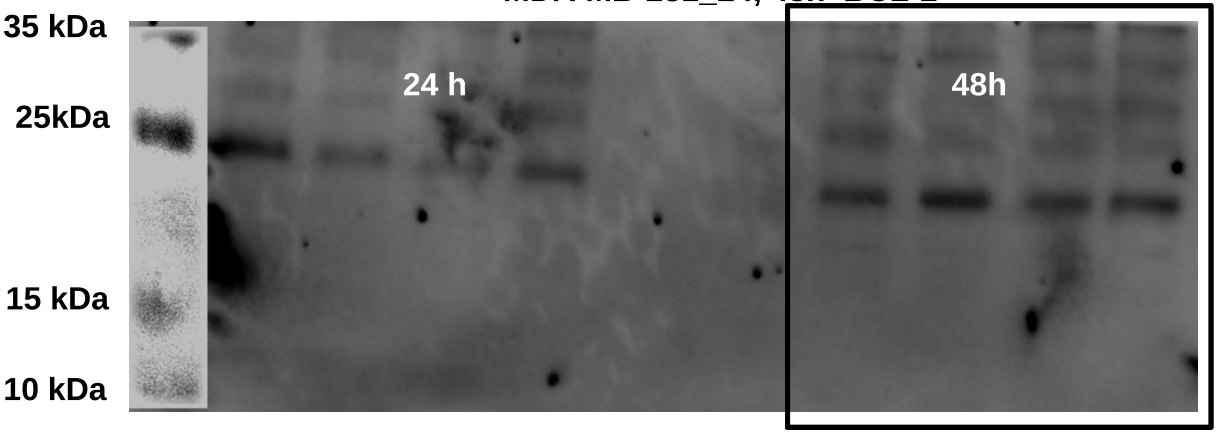

MDA-MB-231_p53

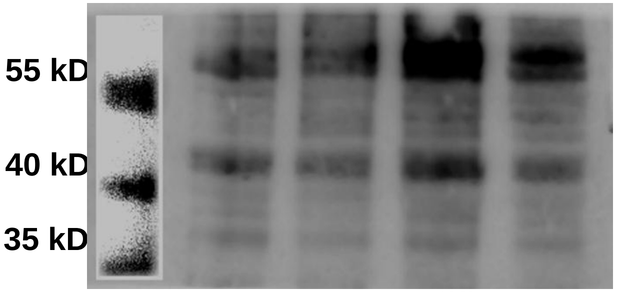

MDA-MB-231_PARP 89

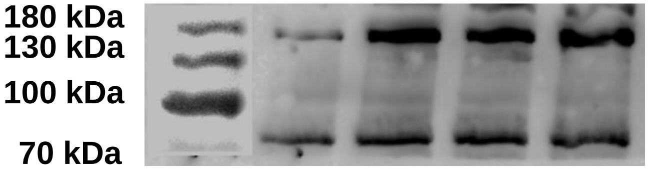

Western blots not shown in Fig. 5 (additional repetitions)

MDA-MB-231_p-BAD Ser112

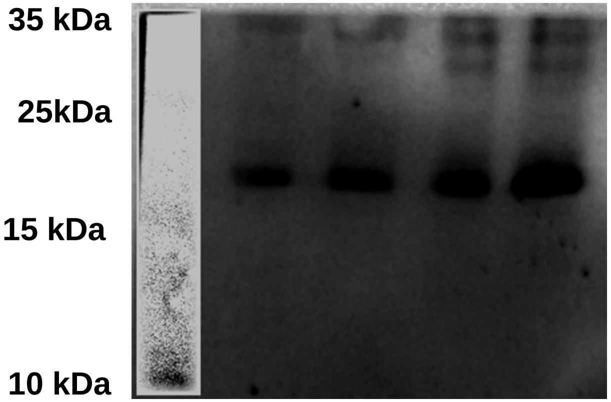

MDA-MB-231_p-BAD Ser112

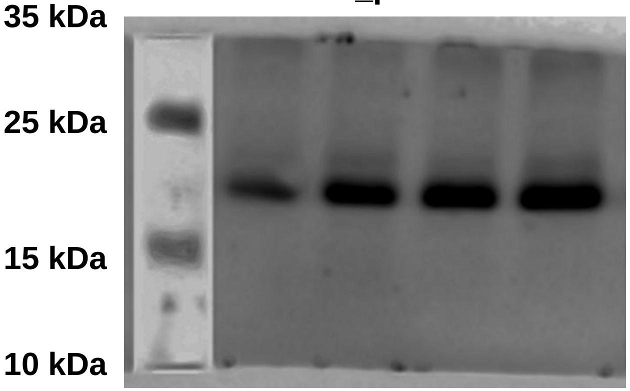

MDA-MB-231_p-BAD Ser112

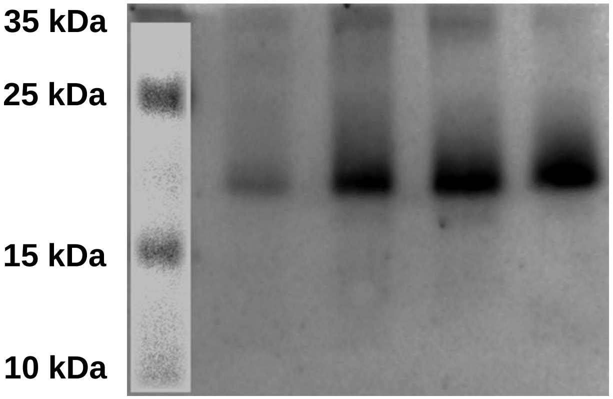

MDA-MB-231_p-BAD Ser112

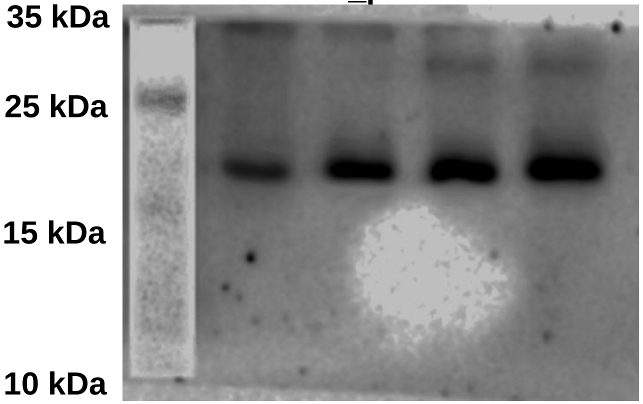

MDA-MB-231_p-BAD Ser112

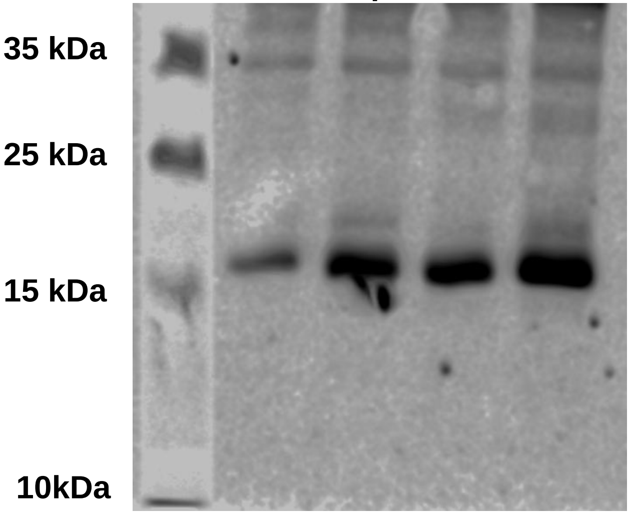

MDA-MB-231_p-BAD Ser112

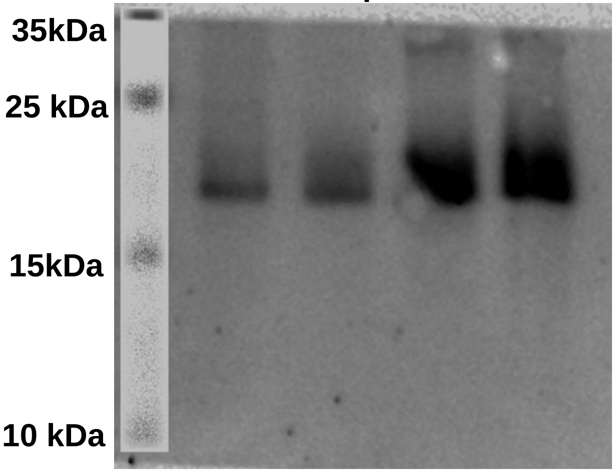

MDA-MB-231_p-BAD Ser 112

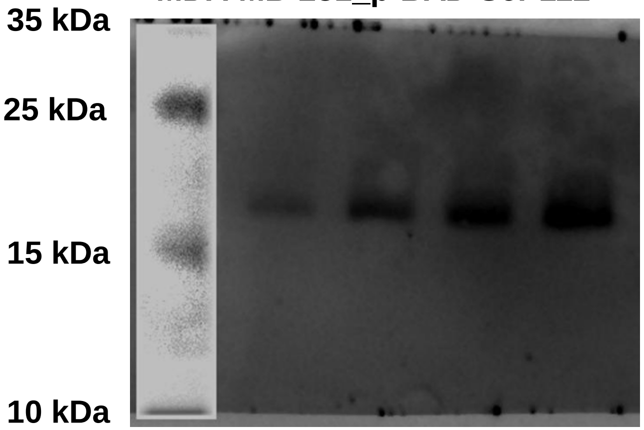

MCF-7

Western blots shown in Fig. 5

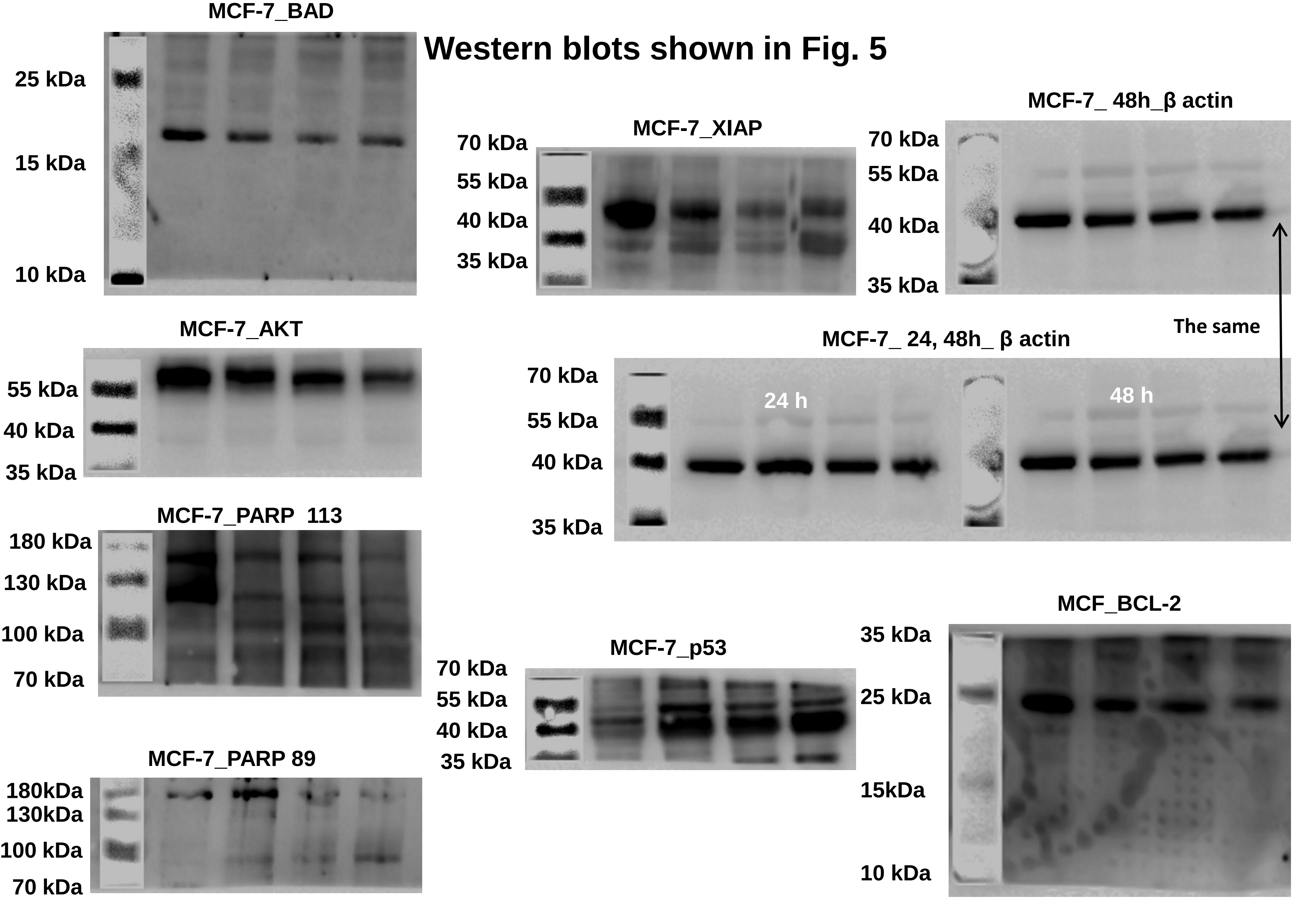

Western blots shown in Fig. 5

MCF-7_β actin_(phospho)

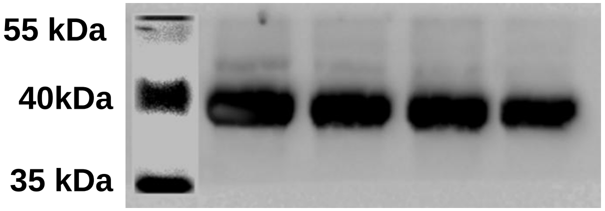

MCF_p-BAD_Ser112

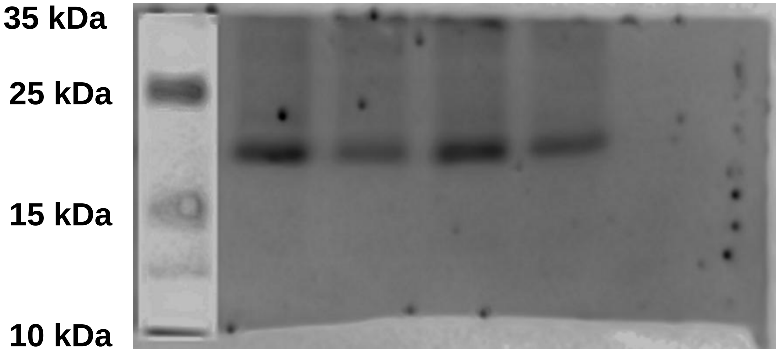

MCF-7_p-AKT1_Ser129

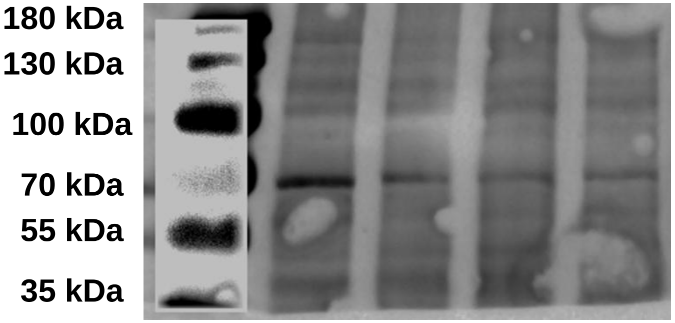

MCF-7_p-p53_Ser15

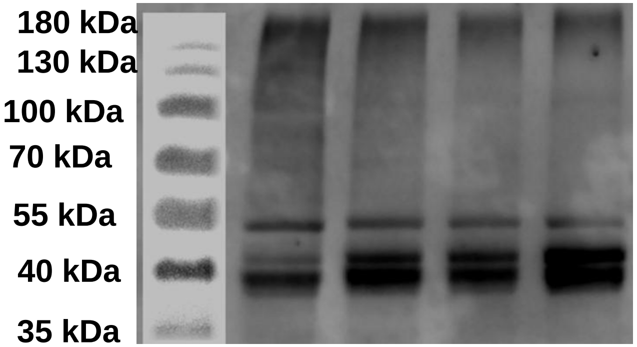

MCF-7_p-p53_Ser392

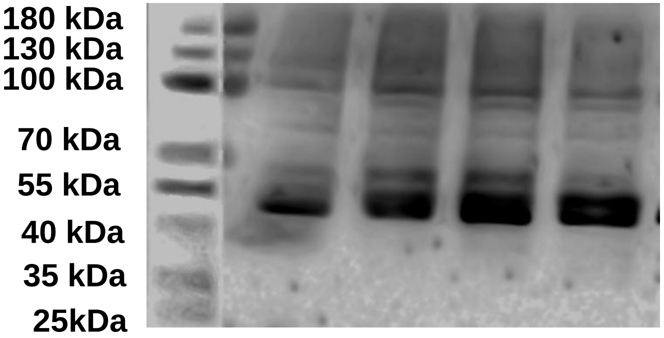

Western blots not shown in Fig. 5 (additional repetitions)

MCF-7_β actin;

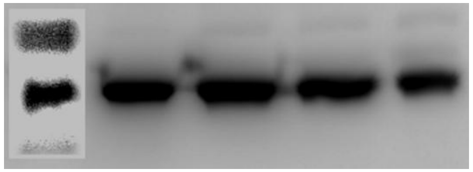

MCF-7_BCL-2

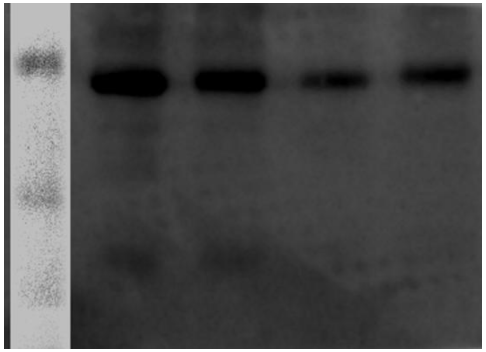

MCF-7_BCL-2

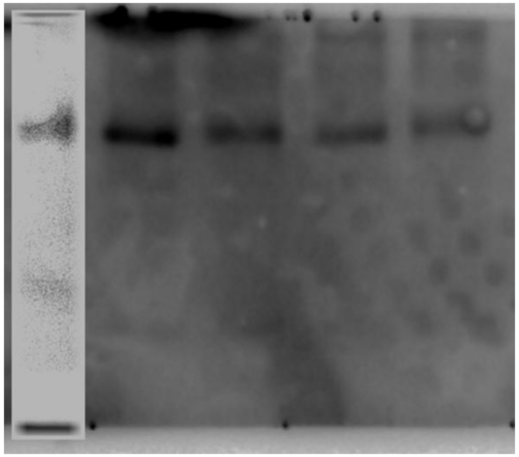

MCF-7_BCL-2

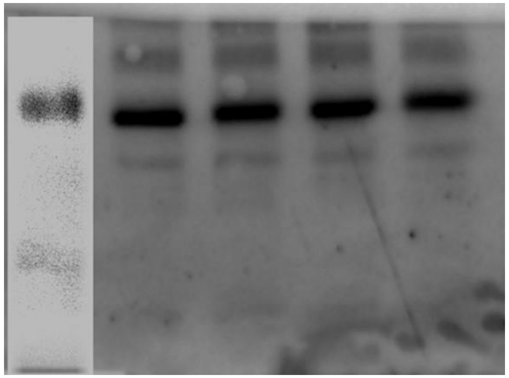

MCF-7_BCL-2

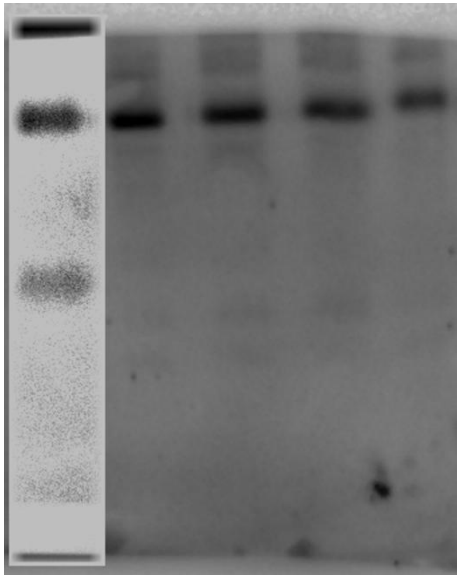

Western blots not shown in Fig. 5 (additional repetitions)

MCF-7_XIAP

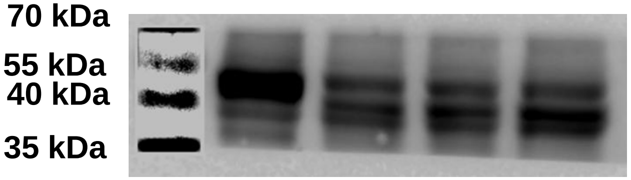

MCF-7_XIAP

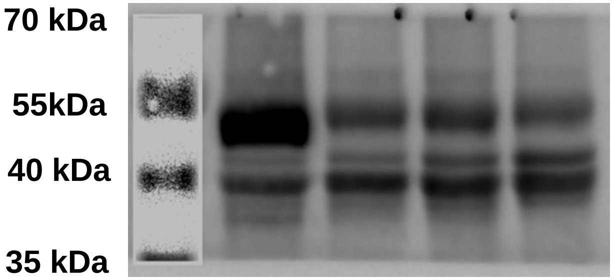

MCF-7_XIAP

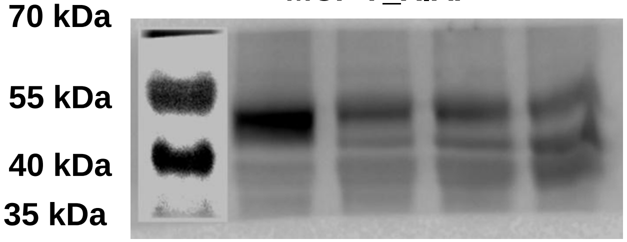

MCF-7_p 53

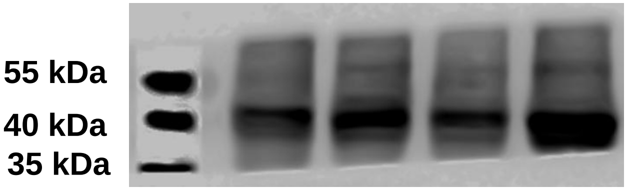

MCF-7_BAD

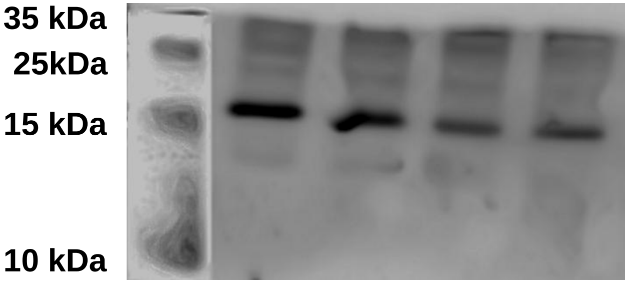

MCF-7_BAD

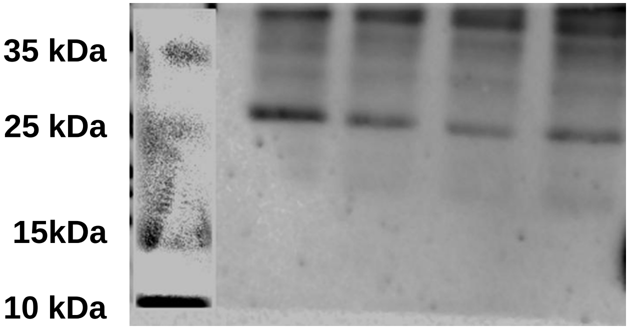

MCF-7_BAD

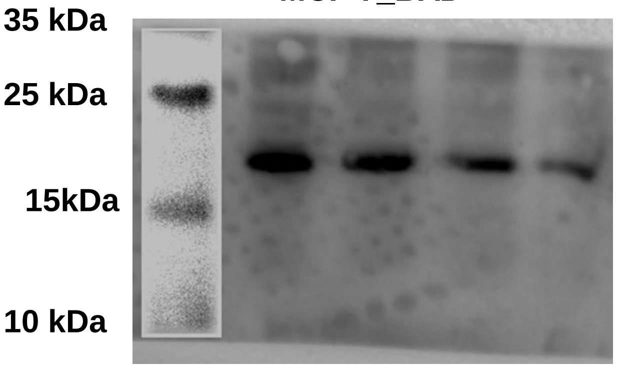

Western blots not shown in Fig. 5 (additional repetitions)

MCF-7_β actin_(phospho)

55 kDa
40 kDa
35 kDa

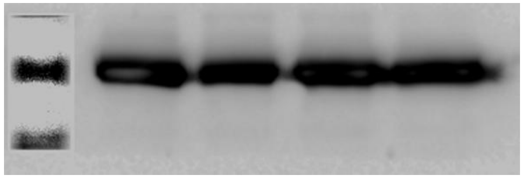

MCF_p-BAD_Ser112

25 kDa

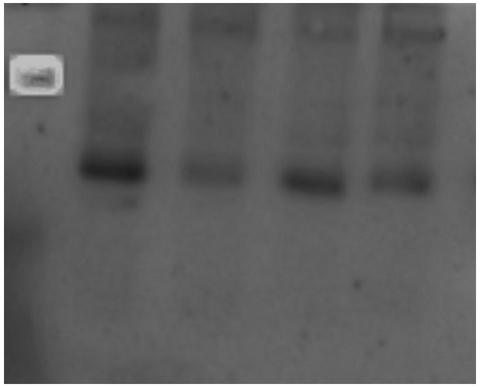

MCF-7_p-BAD_Ser112

25 kDa
15 kDa
10 kDa

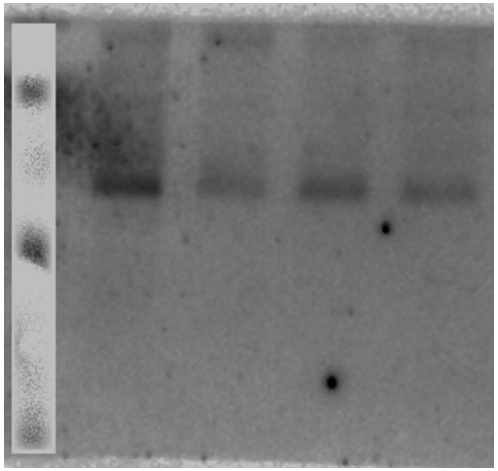

MCF-7_AKT1

55 kDa
40 kDa
35 kDa

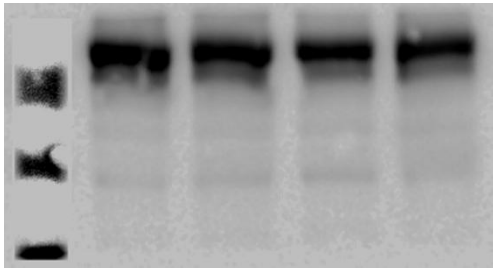

MCF-7_AKT1

70 kDa
55 kDa
40 kDa
35kDa

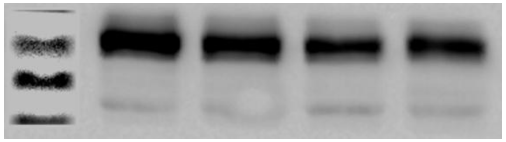

MCF-7_p- p53_Ser392

180 kDa
130 kDa
100 kDa
70 kDa
55 kDa
40 kDa
35 kDa

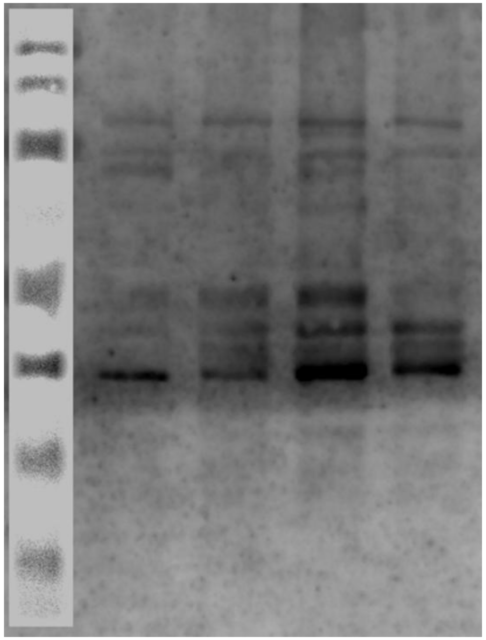

SK-BR-3

Western blots shown in Fig. 5

SK-BR_ 48h_β actin

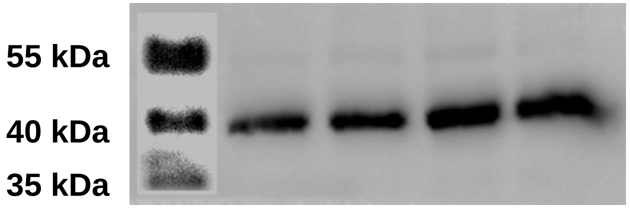

SK-BR-3_p53

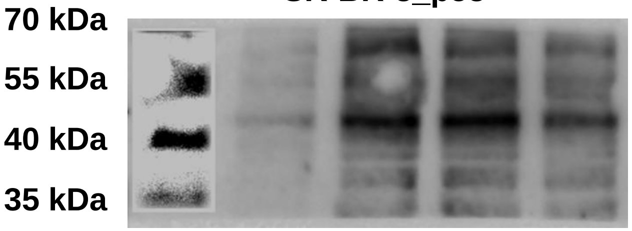

SK-BR-3_XIAP

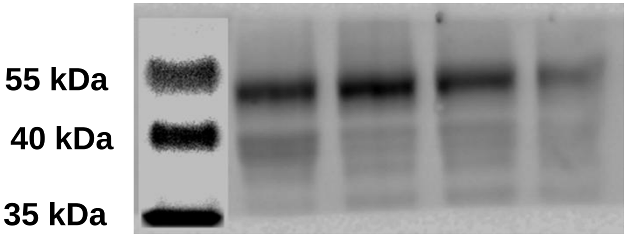

SK-BR-3_PARP 113

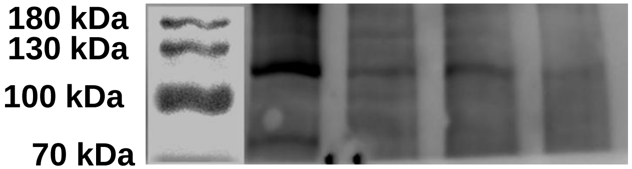

SK-BR-3_PARP 89

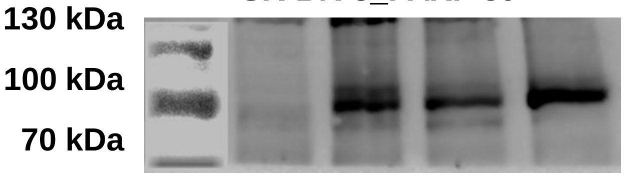

SK-BR-3_AKT1

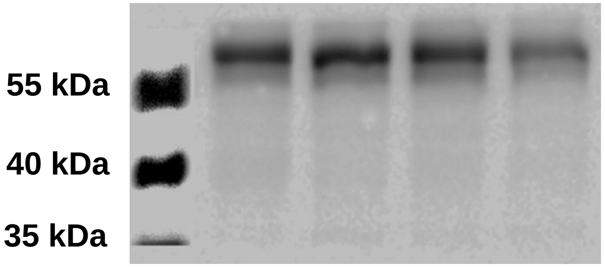

SK-BR-3_BAD

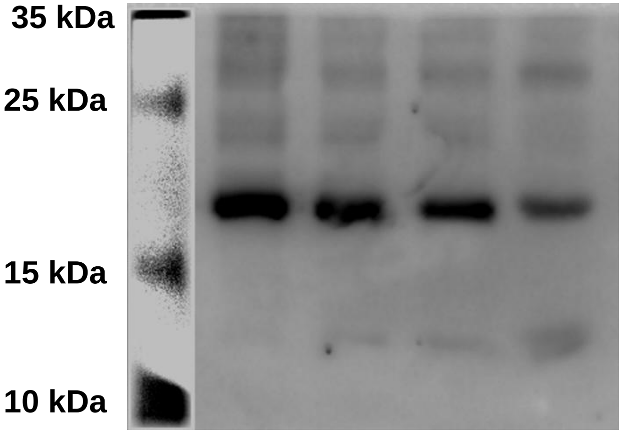

SK-BR-3_BCL-2

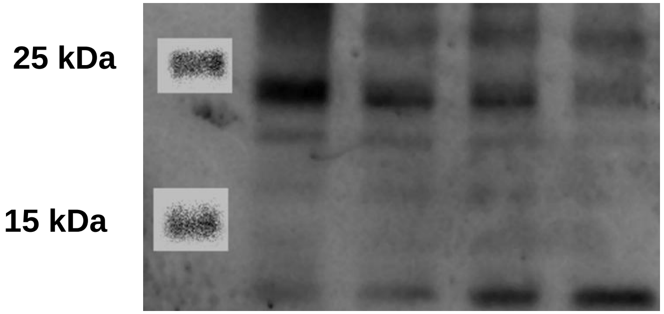

Western blots shown in Fig. 5

SK-BR-3_β actin

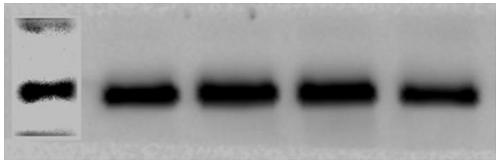

SK-BR-3_p-AKT1_Ser129

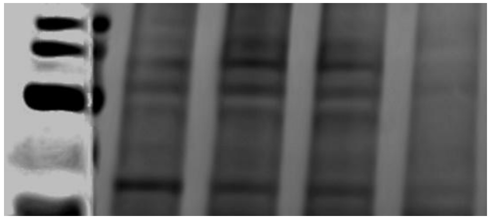

SK-BR-3_p-BAD_Ser112

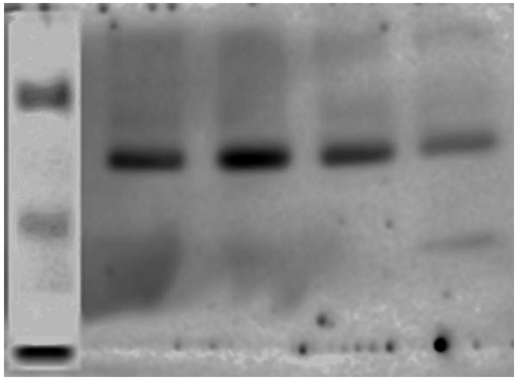

SK-BR-3_p-p53_Ser15

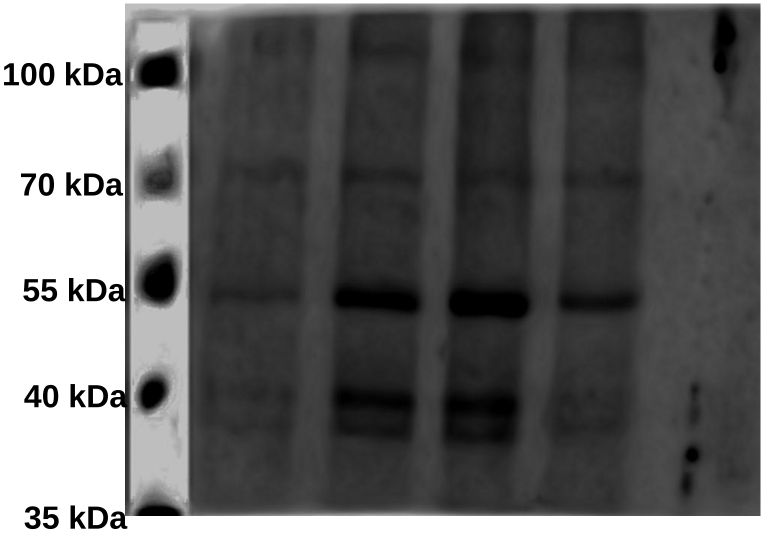

SK-BR-3_p-p53_Ser392

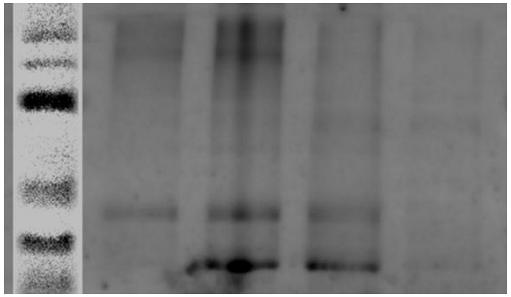

Western blots not shown in Fig. 5 (additional repetitions)

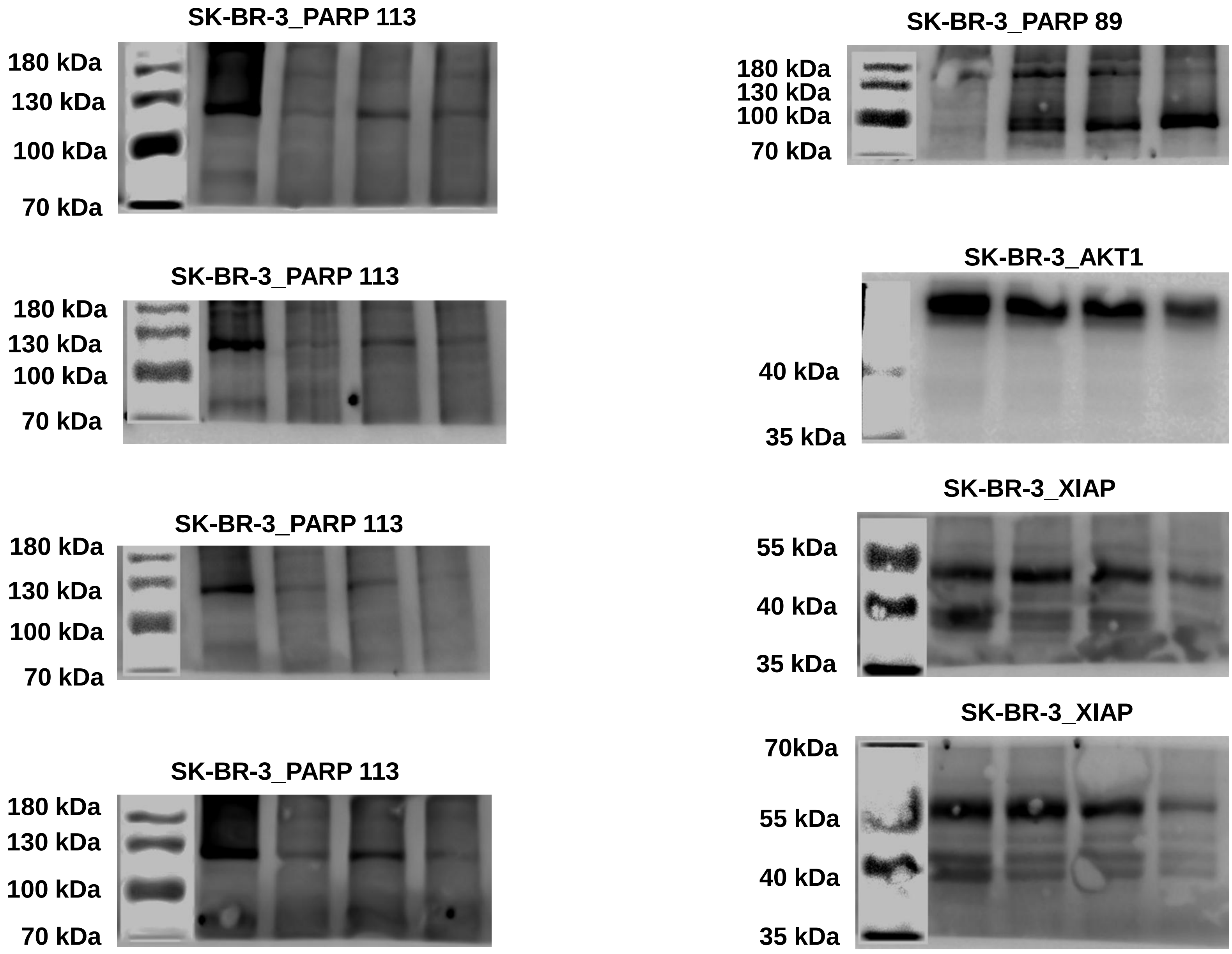

Western blots not shown in Fig. 5 (additional repetitions)

SK-BR-3_ β actin (phospho)

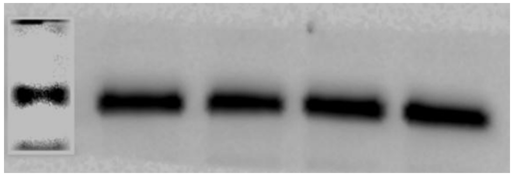

SK-BR-3_β actin (phospho)

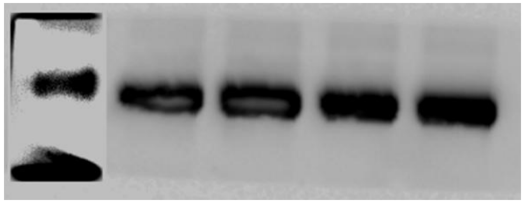

SK-BR-3_p-AKT1_Ser129

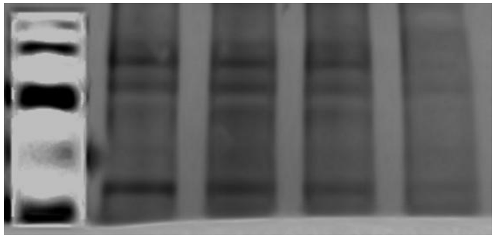

SK-BR-3_p-AKT1_Ser129

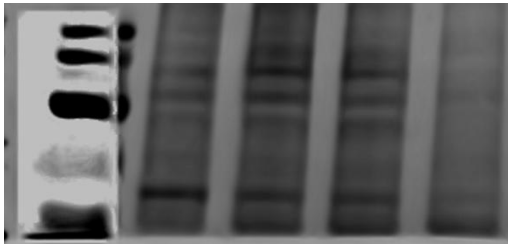

SK- BR-3_p-p53_Ser15

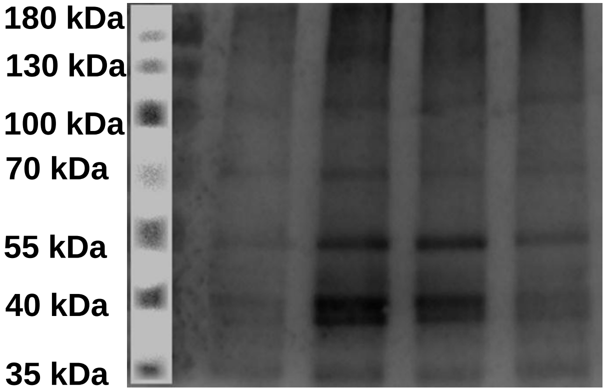

SK-BR-3_p-BAD_Ser112

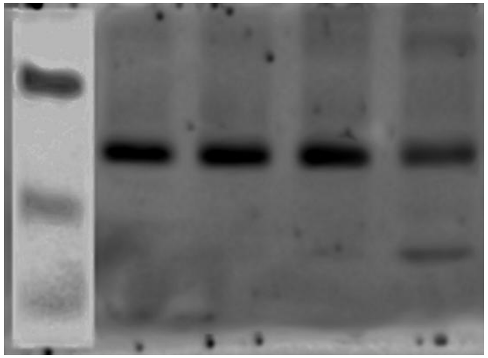

Supplement: Supplementary file 1 — Additional file 1. [file 12885_2022_10156_MOESM1_ESM.pdf]
